# Enhancing substance use detection in clinical notes with large language models

**DOI:** 10.1016/j.drugalcdep.2025.112888

**Published:** 2025-09-30

**Authors:** Fabrice Harel-Canada, Anabel Salimian, Brandon Moghanian, Sarah Clingan, Allan Nguyen, Tucker Avra, Michelle Poimboeuf, Ruby Romero, Arthur Funnell, Panayiotis Petousis, Michael Shin, Nanyun Peng, Chelsea L. Shover, David Goodman-Meza

**Affiliations:** a Computer Science Department, University of California, Los Angeles, 404 Westwood Plaza Suite 277, Los Angeles, 90095, CA, USA; b Semel Institute for Neuroscience and Human Behavior, University of California, Los Angeles, 760 Westwood Plaza, Los Angeles, 90024, CA, USA; c University of California, Los Angeles, 200 Medical Plaza Suite 365C, Los Angeles, 90024, CA, USA; d Integrated Substance Abuse Programs, University of California, Los Angeles, 10911 Weyburn Ave, Ste. 200, Los Angeles, 90024, CA, USA; e David Geffen School of Medicine, University of California, Los Angeles, 10833 Le Conte Ave, Los Angeles, 90095, CA, USA; f Division of General Internal Medicine and Health Services Research, University of California, Los Angeles, 1100 Glendon Ave STE 850, Los Angeles, 90024, CA, USA; g Clinical and Translational Science Institute, University of California, Los Angeles, 924 Westwood Blvd Suite 420, Los Angeles, 90024, CA, USA; h Department of Geography, University of California, Los Angeles, 1255 Bunche Hall, Los Angeles, 90095, CA, USA; i Kirby Institute, University of New South Wales, Wallace Wurth Building (C27), Cnr High St & Botany St, UNSW, Sydney, 2052, NSW, Australia

**Keywords:** Substance use, Drug use, People who inject drugs, NLP, Natural language processing

## Abstract

Identifying substance use behaviors in electronic health records (EHRs) is challenging because critical details are often buried in unstructured notes that use varied terminology and negation, requiring careful contextual interpretation to distinguish relevant use from historical mentions or denials. Using MIMIC-III/IV discharge summaries, we created a large, annotated drug detection dataset to tackle this problem and support future systemic substance use surveillance. We then investigated the performance of multiple large language models (LLMs) for detecting eight substance use categories within this data. Evaluating models in zero-shot, few-shot, and fine-tuning configurations, we found that a fine-tuned model, Llama-DrugDetector-70B, outperformed others. It achieved near-perfect F1-scores (≥ 0.95) for most individual substances and strong scores for more complex tasks like prescription opioid misuse (F1=0.815) and polysubstance use (F1=0.917). These findings demonstrated that LLMs significantly enhance detection, showing promise for clinical decision support and research, although further work on scalability is warranted.

## 1. Introduction

Identifying persons who use drugs and understanding their related behaviors are critical for improving patient care. In electronic health records (EHRs), the detailed nuances of substance use are primarily documented within free-text notes, a domain of knowledge confined mainly to the direct care providers who interact with patients daily (Modi et al., 2022; King et al., 2014). While EHRs contain a wealth of data (Menachemi and Collum, 2011), this crucial information regarding substance use and related issues presents a significant challenge for researchers, hospital administrators, and public health agencies seeking to monitor broader usage trends and inform policy (Velupillai et al., 2018; Tayefi et al., 2021). The current landscape often leaves these higher-level stakeholders operating without a comprehensive, aggregated view of substance use patterns within their populations.

Natural language processing (NLP) offers a promising solution to bridge this gap by extracting actionable insights from the vast amounts of unstructured text data in EHRs (Wang et al., 2012; Assale et al., 2019; Mahbub et al., 2025). As a subfield of artificial intelligence, NLP focuses on developing algorithms to understand and analyze human language (Khurana et al., 2023). These techniques have been applied to various tasks, such as classifying clinical notes, extracting patient information (Wang et al., 2015), and screening for potential future substance use (Alemi et al., 2018). In the domain of substance use disorders, NLP has demonstrated effectiveness in detecting opioid misuse (Blackley et al., 2020; Lingeman et al., 2017; Carrell et al., 2015), identifying people who inject drugs (PWID) (Goodman-Meza et al., 2022b), and recognizing substances involved in overdoses (Goodman-Meza et al., 2022a). By transforming these detailed clinical notes into analyzable data, NLP could be used to monitor usage trends, allocate resources effectively, and develop targeted interventions for at-risk individuals.

Recent advances in NLP have led to the development of two prominent types of models: BERT-style encoders (Devlin et al., 2019; Lee et al., 2020; Alsentzer et al., 2019; Huang et al., 2019) and GPT-style decoders (Brown et al., 2020; Achiam et al., 2023; Dubey et al., 2024), which have significantly improved our ability to process and generate human language (Zhao et al., 2023). These large language models (LLMs), extensively pre-trained on vast text datasets, can perform a wide range of tasks, often with little (few-shot) to no (zero-shot) task-specific data. This flexibility makes them particularly attractive for scenarios where labeled data is scarce, such as in clinical domains focused on substance use (Barenholtz et al., 2020; Bell et al., 2013; Ni et al., 2021; Riddick and Choo, 2022; Hylan et al., 2015). Despite their proven value, their applications to substance use detection in unstructured EHRs are under-explored. Therefore, this study aimed to evaluate the performance of contemporary zero-shot and few-shot NLP models in identifying substance use and related features from unstructured text in EHRs.

## 2. Methods

### 2.1. Dataset

We performed a retrospective study to evaluate the performance of different LLMs at identifying reported substances used by patients within unstructured text from EHRs. Our analysis used records from MIMIC-III (2001–2012) (Johnson et al., 2016), focused on intensive care unit (ICU) patients, and MIMIC-IV (2008–2019) (Johnson et al., 2023), which includes both ICU and emergency department patients. The clinical notes therefore represent a high-acuity, inpatient population, rather than an outpatient or overdose-specific setting. As this analysis involved only de-identified, publicly available data, the University of California, Los Angeles Institutional Review Board (IRB) determined this study to be exempt from IRB oversight.

### 2.2 Substance classes

Given the prevalence of polysubstance use, we framed this task as a multi-label text classification problem to capture concurrent use. In our setup, the input was a medical note containing potential references to substance use. The output was a binary vector indicating the presence or absence of eight items of interest: heroin, cocaine, methamphetamine, illicit use of prescription opioids and benzodiazepines, cannabis, injection drug use (IDU), and general drug use (Any). Although fentanyl was also considered an item of interest, it was excluded from the final set due to its infrequency in our dataset.

#### 2.2.1. Human annotation

We identified 1151 notes containing keywords relevant to the eight drug classes. This pool of notes was first identified electronically using a broad keyword search designed to maximize recall across the target categories. Each of these notes was then reviewed in detail by our annotation team to ensure accuracy, so the final annotated corpus reflects full manual verification even though the initial screening step was automated. Five team members (AS, BM, SC, AN, TA) were trained to recognize both explicit and nuanced mentions of substances used based on a pre-specified annotator guide (Appendix A). For instance, while prescription opioids were frequently mentioned benignly in medical notes, identifying illicit use required careful contextual understanding. Annotators highlighted spans of one or more words and classified them under one of the drug classes. Each text span was assigned a single drug class, although multiple spans could be annotated within the same sentence or note.

All team members annotated the same set of 100 notes (10%), and kappa statistics (Cohen, 1960) were computed to assess inter-annotator agreement. Upon achieving a kappa score above 0.80 for each class, indicating strong agreement (McHugh, 2012), annotators proceeded to single-annotate a subset of the remaining notes. A final team member (AS) then reviewed all annotations for accuracy.

#### 2.2.2. Data preprocessing

Due to the significant length of the original medical notes, which posed challenges for standard NLP techniques, we employed span-level annotations. This method breaks down the text into meaningful segments (spans) for individual analysis. While this initially allowed consideration of token classification models—assigning classes like drug names to each word (Liu et al., 2021; Polignano et al., 2021), the potential computational intensity of classifying every token led us to adopt a different strategy. We reframed the task as multi-label sentence classification, assigning multiple relevant labels (e.g., identifying both “cocaine” and “cannabis”) to each sentence, thereby capturing diverse information more efficiently than word-by-word analysis.

By tokenizing the annotated medical notes into sentences, we compiled a dataset of 274,602 rows, with only 3948 containing drug mentions. To evaluate zero and few-shot model performance, we created class-balanced dataset splits for training, validation, and test splits with 10%:10%:80% data points, respectively. We distributed all instances of these classes across the dataset splits. However, some skew was inevitable due to the prevalence of these substances. For example, heroin and cocaine mentions were more common and are overrepresented relative to other drug classes, such as methamphetamine. This final, processed collection of annotated sentences, along with their corresponding multi-label classifications and train/validation/test splits, constitutes the novel DrugDetection dataset created for this study.

### 2.3. Detection models

We evaluated a range of NLP models for detecting substance use in medical notes. We considered key dimensions such as model architecture, pre-training specialization, and availability in a comprehensive, concurrent analysis. We compared smaller, more efficient BERT-style encoders with larger GPT-style decoders (commonly known LLMs), each offering distinct benefits in terms of processing speed, computational demands, and task performance. BERT-style encoders capture context bidirectionally, making them effective for understanding nuanced medical language, while GPT-style decoders process text sequentially, optimizing for fluent generation but lacking full bidirectional context.

We also assessed the impact of domain-specific pre-training, particularly in the medical field, to determine whether specialized training enhances the models’ ability to detect substance use accurately. Additionally, our selection included both open-source and proprietary models to address critical concerns like cost, accessibility, transparency, and privacy—factors that are especially important in medical applications. To further explore the impact of few-shot fine-tuning, we created Llama-DrugDetector in both 8B and 70B parameter versions, optimized for substance use identification tasks in electronic health records using only a limited number of examples (*n* = 804). A summary of the models we studied is provided in Table 1, with additional details on each model available in Appendix B, and a description of our fine-tuning process in Appendix C.

### 2.4. Detection pipelines

We developed custom detection pipelines to evaluate the performance of various model paradigms in detecting reported substances used and IDU.

For the BERT-style encoders, zero-shot analysis was not advisable because the added classification layers require at least some tuning to map inputs to outputs meaningfully. Therefore, we focused on few-shot fine-tuning, both with and without additional medical domain pre-training. This process involves updating the model weights based on errors observed in a small set of examples.

In addition to few-shot fine-tuning, LLMs support in-context learning (ICL) (Dong et al., 2024). In this setting, the LLMs learn the task directly from a few examples provided within the context of the prompt, without requiring updates to the model itself. We evaluated the LLMs under zero-shot (no examples) and few-shot (few examples) configurations randomly drawn from the validation split of the dataset. This allowed us to characterize the performance of few-shot fine-tuning combined with few-shot ICL. For locally hosted LLMs, we implemented our prompting pipelines using guidance (Lundberg et al., 2022), a constrained decoding framework that enforces well-formed outputs. Since guidance requires access to token probabilities to function optimally and proprietary LLM providers do not provide this data, we instead implemented a separate pipeline for the GPT family of models in langchain (Chase, 2022).

### 2.5 Evaluation metrics

We compared the performance of all models to identify the best combinations of fine-tuning and prompting strategies. Using the held-out test split (n = 6443) of the DrugDetection dataset, we calculated diagnostic metrics including F1-score, accuracy, sensitivity (i.e., recall), positive predictive value (i.e., precision), specificity, and negative predictive value. The F1-score, which balances positive predictive value and sensitivity, is particularly useful in cases with an uneven distribution of positive and negative instances.^1^ We calculated 95% confidence intervals (CIs) via bootstrapped resampling. We bootstrapped the testing set with replacement 1000 times, running each test on 100 samples and calculating diagnostic metrics for each resample. The 2.5th and 97.5th percentiles were reported as the lower and upper ends of the CI, respectively, and the 50th percentile as the mean. Lastly, we performed a manual error analysis of the false-positive and false-negative predictions from the best-performing NLP model. All statistical analyses were performed using Python 3.12 software.

### 2.6. Error analysis

Lastly, we conducted several rounds of error analysis to identify specific weaknesses in model performance, categorizing and quantifying the most common errors. Based on these analyses, we iteratively refined our prompts to address these issues. The final prompt template we used for all LLMs is available in Appendix D.

### 2.7. ICD code analysis

To assess whether structured diagnostic codes adequately capture documented substance use, we linked our human-annotated DrugDetection labels to discharge diagnoses in MIMIC-III and MIMIC-IV. We evaluated International Classification of Diseases (ICD-9-CM/ICD-10) (National Center for Health Statistics, 2011; World Health Organization, 2016) codes as proxies for substance use identification. Each substance category was mapped to corresponding ICD-9 code lists and ICD-10 code prefixes (Appendix Table 14). Injection drug use could not be evaluated as no dedicated ICD-9/10 codes exist in standard mappings. The “Any” category represents the union across all substance categories.

At the patient level, we classified a category as ICD-positive if any corresponding code appeared during the admission. Using human annotations as the reference standard, we computed sensitivity, specificity, and F1-score for each category.

### 2.8. Racial fairness analysis

To assess potential performance disparities between demographic racial groups, we evaluated our best-performing model (Llama-DrugDetector-70B) by structured race categories available in the dataset. We first aggregated fine-grained race labels into broader groups (e.g., “HISPANIC/LATINO – PUERTO RICAN” → “Hispanic/Latino”). To ensure stable estimates, we evaluated only race–substance pairs with ≥5 patients positive for that substance in the test set. After filtering, 29 of 32 possible pairs (90.6%) remained viable for analysis (4 race groups × 8 substances). The “Other” category grouped non-specific and “unknown” labels.

We quantified between-group performance using F1 disparity, defined for each substance as: (1) the absolute difference between maximum and minimum race-specific F1 scores, and (2) the relative ratio (minimum/maximum). These metrics were compared against the commonly used 80% rule-of-thumb threshold for fairness assessment (EEOC, DOJ, DOL, CSC, 1978; Equal Employment Opportunity Commission, 1979; Watkins and Chen, 2024).

## 3. Results

For simplicity of presentation, we focus on overall performance by model aggregated across all drug classes. Complete performance breakdowns by metric can be found in the Appendix E.

### 3.1. Dataset statistics

The text inputs in the DrugDetection dataset average 17.2 words each. These were derived from the original notes, which, prior to sentence tokenization, were substantially longer, averaging 239 sentences and about 2840 words per note.

Table 2 summarizes key statistics for each dataset split and for the full DrugDetection dataset. To better evaluate the precision of substance detection systems, we included medical notes unrelated to substance use (see the “None” column), making up roughly 50% of the overall dataset. Additional details, including substance co-occurrence patterns, are provided in Appendix F.

### 3.2. BERT-style model evaluation

Table 3 compares four BERT-style decoder models on our DrugDetection dataset, including the general-purpose bert-base-uncased and three bio-clinical variants. Contrary to conventional expectations, the base model demonstrated competitive performance, achieving the highest accuracy (0.691, 95% CI: 0.679–0.701) and specificity (0.962, 95% CI: 0.959–0.964) among all models, along with superior precision (0.334, 95% CI: 0.290–0.378). While ClinicalBERT attained the highest F1-score (0.308, 95% CI: 0.298–0.318) and sensitivity (0.363, 95% CI: 0.356–0.371), its performance margins over the base model remain narrow, with overlapping confidence intervals in most metrics. The bio-clinical models showed mixed results: Bio_ClinicalBERT achieved marginally better accuracy (0.689 vs. 0.683) than ClinicalBERT but lower sensitivity, while biobert-v1.1 in F1-score (0.276) and precision (0.265). All models exhibit strong negative predictive value (NPV ≥ 0.966) and specificity (≥ 0.954), indicating robust identification of true negatives, but struggle with positive case detection (sensitivity ≤ 0.363).

### 3.3. Zero-shot LLM model evaluation

Table 4 compares zero-shot performance of Llama-3 variants with and without bio-clinical adaptation. The base Llama-3.1–8B-Instruct maintained superior performance among 8B models, achieving the highest F1-score (0.706) and accuracy (0.716), though domain-adapted Llama3-OpenBioLLM-8B demonstrated exceptional precision (0.728) and specificity (0.987). Notably, Llama3-Med42–8B showed dramatic sensitivity (0.968) at the cost of precision (0.499), suggesting over-detection tendencies. For 70B models, the generalist DeepSeek-R1-Distill-Llama-70B remained dominant with peak F1-score (0.871) and accuracy (0.860). All models exhibited strong negative predictive value (NPV ≥ 0.946) and specificity (≥ 0.833), mirroring patterns observed in BERT-style detectors (Table 3), but LLMs demonstrated substantially higher sensitivity (recall ≥0.680 vs. ≤0.363 in BERT models). While domain adaptation showed potential in specific metrics (*e.g*., Llama3-OpenBioLLM-8B’s precision outperformed base models by 15.8%), the general superiority of base architectures motivated our selection of DeepSeek-R1-Distill-Llama for few-shot fine-tuning.^2^

### 3.4. Few-shot fine-tuning and few-shot ICL

**Fig. 1.**
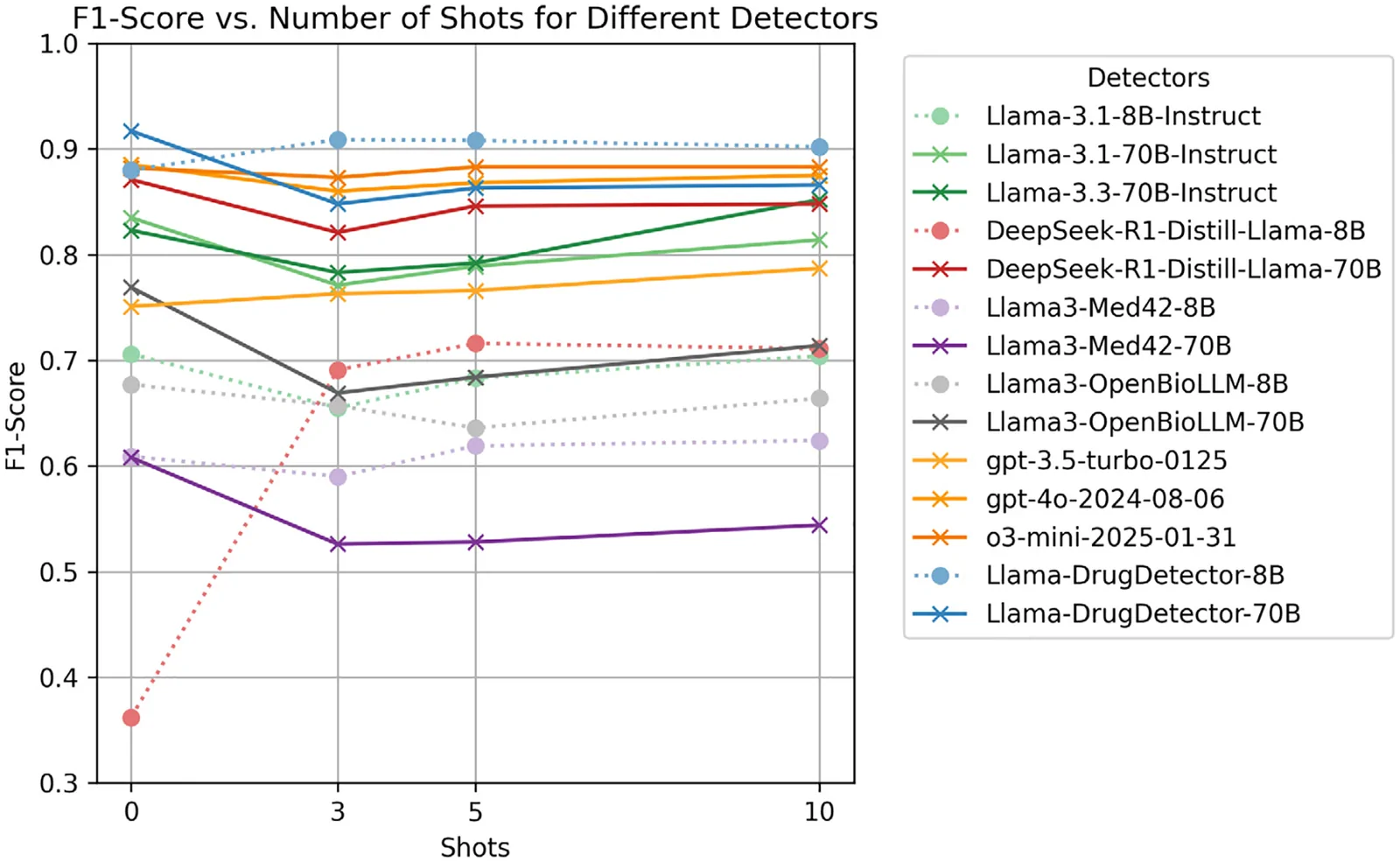
Overall F1-score per model when given N-Shot examples in the prompt, with models grouped by family and colored accordingly. Dotted lines indicate 8B models, whereas solid lines denote 70B or models of unspecified size. Across detectors, the maximum improvement in F1-Score was 35.4% (DeepSeek-R1-Distill-Llama-8B), and the average improvement was ~ 1.3%.

Fig. 1 illustrates the impact of incorporating few-shot examples within the context of the prompt before the main classification task. In 6 out of 15 instances, including few-shot examples led to an enhancement in F1-score compared to the zero-shot baseline. Notably, DeepSeek-R1-Distill-Llama-8B exhibited a substantial 35.4% improvement. On average, few-shot in-context learning resulted in a ~ 1.3% performance boost. Additionally, we observed that combining few-shot fine-tuning with few-shot ICL yielded significant benefits. Our best overall model, Llama-DrugDetector-70B, achieved the highest zero-shot performance (91.9%) but did not benefit from few-shot examples. However, Llama-DrugDetector-8B did benefit from few-shot examples and was the highest performing 8B model. This demonstrated the potential of few-shot ICL in enhancing model accuracy, particularly when integrated with fine-tuning strategies.

### 3.5. Comparing best overall performance

Table 5 compares model performance in the challenging polysubstance detection setting, where models must simultaneously identify all relevant drug classes (accuracy requires perfect multi-label classification). Our fine-tuned Llama-DrugDetector-70B achieved the highest F1-score (0.917), while its 8B counterpart (Llama-DrugDetector-8B) demonstrated exceptional accuracy (0.939) and specificity (0.994), suggesting particular strength in avoiding false positives across multiple substance categories. Proprietary models without fine-tuning show mixed performance: o3-mini-2025-01-31 achieved peak specificity (0.994) and competitive precision (0.893) but trailed in F1-score (0.883 vs. 0.917). The all-classes-correct requirement exacerbated architectural disparities, with 70B LLMs achieving 2.97× higher F1-scores than the best-performing BERT-style variants (0.917 vs. 0.308). Finally, fine-tuned open-source models consistently outperformed proprietary counterparts in critical metrics — Llama-DrugDetector-70B surpassed gpt-4o-2024-08-06 in F1-score (0.917 vs. 0.885).

### 3.6. Comparing substance-specific performance

The F1 performance for detecting substances individually is presented in Table 6, with similar tables for other metrics available in Appendix E. Detection complexity and performance varies substantially by substance class, and different models possess different strengths. For example, prescription opioid misuse proved most challenging (best F1: 0.830, Llama-DrugDetector-8B), while all other classes exhibited perfect or near-perfect detection (*geq* 0.95). Heroin detection peaked with o3-mini-2025-01-31 (0.985), while cocaine identification was strongest in Llama-DrugDetector-70B (0.994). Methamphetamine detection reached perfection (F1: 1.000) in Llama-3.3–70B-Instruct, though its narrow applicability is evident from lower scores in prescription opioid misuse (0.655).^3^

### 3.7. Error analysis

Table 7 presents the final error analysis for the best-performing model, our fine-tuned DrugDetector-70B. The most common issue was insufficient evidence, where the model made assumptions unsupported by the text — such as inferring heroin use from injection drug use (n = 126) or assuming illicit use of prescription opioids (n = 103). Another major category was simple failures to identify substances (n = 113), including missed mentions or negated statements. Additional errors stemmed from confusion, such as hallucinating drug use (n = 30), misattributing substance use to the patient rather than a family member, or misreading typos.

### 3.8. Comparison with ICD-based detection

Structured ICD codes captured only a fraction of substance use documented in clinical narratives. The macro-average sensitivity across specific substances was 38.1%, while the broadest category “Any” achieved 58.8% sensitivity (specificity 91.4%, F1 71.4%). Among patients with annotated substance use (n=690), only 406 (58.8%) had corresponding ICD codes.

Detection rates varied markedly by substance. ICD codes identified 209 of 382 patients with documented heroin use (54.7% sensitivity), 102 of 237 with cocaine use (43.0%), 14 of 44 with methamphetamine use (31.8%), 26 of 136 with benzodiazepine use (19.1%), 55 of 98 with prescription opioid misuse (56.1%), and 20 of 83 with cannabis use (24.1%). Specificity exceeded 96% for most substances except heroin (87.8%) and prescription opioids (77.4%). F1-scores ranged from 27.7% (prescription opioids) to 60.8% (heroin). Complete results appear in Appendix Table 15.

### 3.9. Performance across racial groups

Analysis of Llama-DrugDetector-70B performance across racial groups revealed minimal disparities. Among 29 evaluable race–substance pairs, the mean absolute F1 difference was 4.5 percentage points. The maximum gap occurred for prescription opioid detection: 7.1 points between Hispanic/Latino patients (F1: 77.6%) and Other/Unknown patients (F1: 70.5%), yielding a relative ratio of 0.91. All relative performance ratios exceeded the 0.80 threshold commonly used in fairness assessments. Detailed breakdowns appear in Appendix Figures 3–4.

## 4. Discussion

This study rigorously evaluated the efficacy of various NLP models, ranging from traditional BERT-style encoders to decoder-only LLMs, in the critical task of detecting substance use mentions within electronic health records. Our findings demonstrate a meaningful shift in the use of NLP for this domain, revealing that even with limited fine-tuning data, contemporary LLMs can achieve remarkable performance, significantly surpassing previous benchmarks that relied on extensive training datasets (Goodman-Meza et al., 2022b; Carrell et al., 2015; Lingeman et al., 2017). Notably, our open-source model, fine-tuned using a few hundred training examples, Llama-DrugDetector-70B,^4^ achieved an F1-score of 0.919 for concurrent polysubstance use, while substance-specific F1 scores ranged from 0.815 for prescription opioid misuse to 0.994 for cocaine use. This exceptional performance, coupled with small per-race F1 gaps (mean 4.5%; max 7.1%) and tighter confidence intervals than gpt-4o-2024-08-06, underscores the potential for enhanced clinical reliability and the feasibility of deploying such tools in real-world healthcare settings. The ability of our model to identify both explicit and contextually nuanced substance references addresses a significant limitation of earlier rule-based systems (Lingeman et al., 2017), offering a more adaptable solution to the evolving landscape of clinical documentation.

A consistent trend throughout our analysis was the superior performance of LLMs over BERT-style encoders across a spectrum of metrics, particularly in the nuanced task of substance-specific detection. This advantage likely stems from the inherent capacity of LLMs to model complex language patterns and capture subtle contextual cues, which are crucial for accurately identifying drug use within clinical narratives (Agrawal et al., 2022; Hu et al., 2024). The observation that open-source LLMs often matched or even exceeded the performance of proprietary models like GPT-4o has significant implications for accessibility and deployment. Importantly, open-source models offer the advantage of being locally hosted, making them more suitable for production use in medical and clinical settings where data privacy is legally and ethically mandated (Salam et al., 2025).

Beyond the immediate clinical applications of improved drug detection, our findings have significant implications for public health surveillance and research. The ability to accurately and efficiently extract information about substance use from EHRs can provide valuable data for monitoring trends in drug use prevalence, which are often difficult to ascertain via traditional methods (Reuter et al., 2021; Keyes et al., 2022; Krawczyk et al., 2022; Lim et al., 2024) and are undercaptured in structured ICD fields (coverage sensitivity 58.8%). By transforming unstructured clinical text into actionable data, our approach can facilitate a more comprehensive understanding of the evolving drug landscape and enable better-informed public health interventions and policy decisions at both the hospital and government levels. The potential for integrating insights from our models with other public health data sources, such as overdose statistics, could further enhance our ability to track and respond to the opioid crisis and other substance use challenges.

The error analysis conducted on our best-performing model, Llama-DrugDetector-70B, provided valuable insights into its remaining weaknesses. The prevalence of errors related to insufficient evidence, failures to detect substances, and confusion between similar terms highlights the challenges inherent in interpreting complex medical language and the need for further refinement in handling contextual nuances. These findings suggest that future work could focus on improving the model’s ability to reason about implicit information, handle negation and subtle linguistic cues, and better distinguish between substances with similar names. Techniques such as incorporating more sophisticated prompt engineering strategies or augmenting the fine-tuning data with examples specifically designed to address these error types might also be beneficial.

## 5. Limitations

This study has several important limitations. First, our findings are based on data from a single medical center in Boston spanning the years 2001–2019, which may limit generalizability. As prior work has shown, different regions of the US exhibit unique drug use profiles that continue to change, further emphasizing the need for models trained on broader datasets (Mattson, 2021; Friedman and Shover, 2023; Shover et al., 2024). Drug use patterns have also evolved over time, and our data largely predates the widespread proliferation of fentanyl and other synthetic drugs in the illicit supply. This timeframe likely explains the low frequency of fentanyl mentions in our dataset. Importantly, during much of this period many clinical centers were not routinely testing specifically for fentanyl, but instead for general opioid classes. In parallel, people who use drugs often report a preferred drug category (e.g., heroin) without knowing the exact composition of the substances consumed, which contributes to under-documentation of fentanyl and other emerging drugs (e.g., xylazine). Together, these factors highlight a structural challenge for text-based detection and underscore the need for models to adapt to evolving drug markets. On a related note, our classification of cannabis as an illicit substance does not account for its legalization for medical (2013) and recreational (2016) use in Massachusetts during the study period. This evolving legal landscape can influence how patients report use and how clinicians document it, a nuance not explicitly captured by our current model.

Second, our modeling approach has limitations regarding its scope and interpretation. We focused on analyzing short medical notes extracted from larger patient profiles. While this approach enabled us to target specific instances of drug-related language, it also introduced the possibility of losing critical context, which could result in misinter-pretation. More generally, our approach does not currently distinguish between a patient’s history of substance use (e.g., in long-term remission) and active, ongoing use. This could lead to an over-estimation of the prevalence of current substance use disorders and highlights an important area for future refinement.

Third, while LLMs offer superior performance in terms of accuracy and language understanding, they demand significantly more computational resources. For instance, BERT-style models process each input in approximately 0.002 s, whereas LLMs require between 0.3 to 20 s, depending on the model. This disparity becomes a substantial challenge when scaling up to process entire patient notes, which contained an average of 239 sentences in our dataset. Expanding the input size to encompass full patient profiles would necessitate advancements in processing speed for LLMs to make such an approach more viable. The significant computational demands of LLMs, contrasted with the faster but more limited BERT-style models, present a clear trade-off between accuracy and efficiency. Future research should explore hybrid models or LLM quantization techniques to address this (Zhou et al., 2024).

## 6. Conclusions

This study establishes that modern LLMs, when combined with few-shot fine-tuning, achieve near-perfect performance in detecting substance use within clinical narratives — surpassing both traditional NLP approaches and proprietary models. Our open-source Llama-DrugDetector-70B attained near-perfect accuracy for most substance classes (except prescription opioid misuse) using only a few hundred training examples, demonstrating that domain-specific performance no longer requires massive labeled datasets or restrictive proprietary systems. These advances carry immediate practical implications: Hospital administrators could deploy such models to flag systemic substance use risks in real time, while public health agencies might leverage them to detect emerging drug trends from unstructured EHR data. However, some challenges remain in minimizing over-interpretation errors (e.g., inferring heroin use from syringe mentions) and optimizing computational costs for clinical workflows. Future work should expand to emerging substances like synthetic opioids (e.g. fentanyl) and explore federated learning frameworks to enhance generalizability across healthcare systems. By open-sourcing our models and benchmarks, we aim to catalyze community-driven improvements in this critical area of clinical NLP.

## Supplementary Material

1

## Figures and Tables

**Table 1:** Summary of selected NLP models categorized by architecture type (Arch.), specialization, and availability. Where possible, we study different sizes of the same model (e.g., Llama-3-Instruct comes in versions with 8B or 70B parameters).

Model name	Arch.	Specialization	Availability
BERT (Devlin et al., 2019)	Encoder	Generalist	Open Source
BioBERT (Lee et al., 2020)	Encoder	Biomedical	Open Source
ClinicalBERT (Huang et al., 2019)	Encoder	Clinical	Open Source
Bio_ClinicalBERT (Alsentzer et al., 2019)	Encoder	Biomedical & Clinical	Open Source
GPT-4o (OpenAI, 2024a)	Decoder	Generalist	Proprietary
Llama-3-Instruct (Dubey et al., 2024)	Decoder	Generalist	Open Source
Llama-3.1-Instruct (Dubey et al., 2024)	Decoder	Generalist	Open Source
Llama-3.3-Instruct (Dubey et al., 2024)	Decoder	Generalist	Open Source
DeepSeek-R1-Distill-Llama (Guo et al., 2025)	Decoder	Generalist	Open Source
MedLlama3 (ProbeMedicalYonseiMAILab, 2024)	Decoder	Biomedical*	Open Source
Llama3-OpenBioLLM (Ankit Pal, 2024)	Decoder	Biomedical*	Open Source
Llama3-Med42 (Christophe et al., 2024)	Decoder	Clinical*	Open Source
Llama-DrugDetector (Ours)	Decoder	Substance Use	Open Source

Asterisks (*) indicate reported domain, but the authors have released no official datasets to confirm content. Llama-DrugDetector is our fine-tuned version of DeepSeek-R1-Distill-Llama.

**Table 2:** Counts of each drug class by split for the DrugDetection dataset. Abbreviations: Methamphetamine Use (Meth.), Benzodiazepine Use (Benzo.), Prescription Opioids Misuse (Rx. Opiods), Injection Drug Use (IDU), and General Drug Use (Any). Total shows the total number of medical notes with zero-to-many substances present in each.

Split	Heroin	Cocaine	Meth.	Benzo.	Rx. Opiods	Cannabis	IDU	Any	None	Total
TRAIN	93	65	9	26	13	13	128	402	402	804
VALIDATION	94	66	9	26	14	14	128	403	403	806
TEST	749	528	72	232	122	121	1041	3143	3300	6443
TOTAL	936	659	90	284	149	148	1297	3948	4105	8053

**Table 3:** Performance of BERT-style decoder models with various types of additional pre-training on bio-clinical data. bert-base-uncased is the base model.

Detectors	F1-Score	Accuracy	Sensitivity (Recall)	Positive predictive value (Precision)	Negative predictive value	Specificity
biobert-v1.1	0.276 (0.271 – 0.281)	0.666 (0.654 – 0.677)	0.329 (0.324 – 0.334)	0.265 (0.240 – 0.300)	0.969 (0.968 – 0.971)	0.954 (0.952 – 0.956)
Bio_ClinicalBERT	0.279 (0.276 – 0.283)	0.689 (0.679 – 0.703)	0.311 (0.307 – 0.315)	0.256 (0.252 – 0.261)	0.966 (0.964 – 0.967)	**0.962** **(0.959 – 0.964)**
bert-base-uncased	0.295 (0.285 – 0.305)	**0.691** **(0.679 – 0.701)**	0.328 (0.322 – 0.335)	**0.334** **(0.290 – 0.378)**	0.969 (0.967 – 0.971)	**0.962** **(0.959 – 0.964)**
ClinicalBERT	**0.308** **(0.298 – 0.318)**	0.683 (0.670 – 0.693)	**0.363** **(0.356 – 0.371)**	0.288 (0.269 – 0.309)	**0.971** **(0.969 – 0.972)**	0.954 (0.952 – 0.957)

**Table 4:** Zero-shot performance metrics of various LLMs with and without additional bio-clinical pre-training, grouped by model size (8B vs. 70B). Llama-3 serves as the base model without targeted domain pre-training. Results indicate that specialized bio-clinical training does not consistently enhance performance for drug detection, motivating the use of a general-purpose model for subsequent task-specific fine-tuning.

Detectors	F1-Score	Accuracy	Sensitivity (Recall)	Positive predictive value (Precision)	Negative predictive value	Specificity
DeepSeek-R1-Distill-Llama-8B	0.362 (0.347 – 0.374)	0.616 (0.606 – 0.629)	0.753 (0.723 – 0.777)	0.285 (0.274 – 0.296)	0.946 (0.944 – 0.949)	0.833 (0.825 – 0.840)
MedLlama3-8B	0.403 (0.392 – 0.413)	0.101 (0.095 – 0.107)	0.928 (0.914 – 0.940)	0.304 (0.293 – 0.314)	**0.988** **(0.985 – 0.992)**	0.628 (0.623 – 0.634)
Llama3-Med42-8B	0.609 (0.586 – 0.628)	0.705 (0.693 – 0.715)	**0.968** **(0.961 – 0.975)**	0.499 (0.479 – 0.516)	0.987 (0.986 – 0.988)	0.928 (0.924 – 0.932)
Llama3-OpenBioLLM-8B	0.677 (0.657 – 0.695)	0.700 (0.689 – 0.712)	0.680 (0.659 – 0.700)	**0.728** **(0.703 – 0.753)**	0.947 (0.944 – 0.949)	**0.987** **(0.986 – 0.988)**
Llama-3.1-8B-Instruct	**0.706** **(0.691 – 0.722)**	**0.716** **(0.706 – 0.724)**	0.941 (0.930 – 0.952)	0.618 (0.602 – 0.637)	0.979 (0.977 – 0.981)	0.957 (0.955 – 0.959)
Llama3-Med42-70B	0.608 (0.591 – 0.626)	0.661 (0.651 – 0.672)	**0.988** **(0.983 – 0.992)**	0.468 (0.451 – 0.485)	0.997 (0.996 – 0.998)	0.908 (0.904 – 0.912)
Llama3-OpenBioLLM-70B	0.769 (0.747 – 0.789)	0.720 (0.710 – 0.730)	0.987 (0.980 – 0.994)	0.659 (0.630 – 0.688)	**0.999** **(0.998 – 0.999)**	0.941 (0.938 – 0.944)
Llama-3.3-70B-Instruct	0.823 (0.813 – 0.832)	0.795 (0.785 – 0.804)	0.985 (0.977 – 0.993)	0.746 (0.729 – 0.761)	0.997 (0.997 – 0.998)	0.967 (0.966 – 0.969)
Llama-3.1-70B-Instruct	0.835 (0.823 – 0.849)	0.828 (0.820 – 0.838)	0.978 (0.967 – 0.986)	0.769 (0.751 – 0.787)	0.997 (0.997 – 0.998)	0.972 (0.970 – 0.974)
DeepSeek-R1-Distill-Llama-70B	**0.871** **(0.857 – 0.883)**	**0.860** **(0.851 – 0.868)**	0.958 (0.948 – 0.970)	**0.831** **(0.814 – 0.847)**	0.991 (0.990 – 0.992)	**0.984** **(0.983 – 0.985)**

**Table 5:** Performance by metric on the held-out test set (n=6443), demonstrating that our fine-tuned models consistently outperform others across nearly all quality dimensions. Parentheses indicate 95% bootstrapped confidence intervals.

Arch	Detector	Shots	F1-Score	Accuracy	Sensitivity (Recall)	Positive predictive value (Precision)	Negative predictive value	Specificity
BERT	biobert-v1.1	0	0.276 (0.271 – 0.281)	0.666 (0.653 – 0.679)	0.329 (0.324 – 0.334)	0.265 (0.240 – 0.300)	0.969 (0.968 – 0.971)	0.954 (0.952 – 0.956)
BERT	Bio_ClinicalBERT	0	0.279 (0.276 – 0.283)	0.689 (0.676 – 0.699)	0.311 (0.307 – 0.315)	0.256 (0.252 – 0.261)	0.966 (0.964 – 0.967)	0.962 (0.959 – 0.964)
BERT	bert-base-uncased	0	0.295 (0.285 – 0.305)	0.691 (0.682 – 0.701)	0.328 (0.322 – 0.335)	0.334 (0.290 – 0.378)	0.969 (0.967 – 0.971)	0.962 (0.959 – 0.964)
BERT	ClinicalBERT	0	0.308 (0.298 – 0.318)	0.684 (0.672 – 0.693)	0.363 (0.356 – 0.371)	0.288 (0.269 – 0.309)	0.971 (0.969 – 0.972)	0.954 (0.952 – 0.957)
LLM	MedLlama3-8B	0	0.403 (0.392 – 0.413)	0.198 (0.190 – 0.209)	0.928 (0.914 – 0.940)	0.304 (0.293 – 0.314)	0.988 (0.985 – 0.992)	0.628 (0.623 – 0.634)
LLM	Llama3-Med42-70B	0	0.608 (0.591 – 0.626)	0.661 (0.646 – 0.672)	0.988 (0.983 – 0.992)	0.468 (0.451 – 0.485)	0.997 (0.996 – 0.998)	0.908 (0.904 – 0.912)
LLM	Llama3-Med42-8B	10	0.624 (0.605 – 0.644)	0.716 (0.705 – 0.727)	0.963 (0.952 – 0.971)	0.502 (0.483 – 0.522)	0.988 (0.987 – 0.989)	0.933 (0.929 – 0.938)
LLM	Llama3-OpenBioLLM-8B	0	0.677 (0.657 – 0.695)	0.699 (0.690 – 0.708)	0.680 (0.659 – 0.700)	0.728 (0.703 – 0.753)	0.947 (0.944 – 0.949)	0.987 (0.986 – 0.988)
LLM	Llama-3.1-8B-Instruct	0	0.706 (0.691 – 0.722)	0.729 (0.720 – 0.740)	0.941 (0.930 – 0.952)	0.618 (0.602 – 0.637)	0.979 (0.977 – 0.981)	0.957 (0.955 – 0.959)
LLM	DeepSeek-R1-Distill-Llama-8B	5	0.716 (0.694 – 0.736)	0.762 (0.752 – 0.770)	0.799 (0.781 – 0.814)	0.670 (0.639 – 0.696)	0.966 (0.963 – 0.968)	0.974 (0.971 – 0.976)
LLM	Llama3-OpenBioLLM-70B	0	0.769 (0.747 – 0.789)	0.787 (0.775 – 0.796)	0.987 (0.980 – 0.994)	0.659 (0.630 – 0.688)	**0.999** **(0.998 – 0.999)**	0.941 (0.938 – 0.944)
LLM	gpt-3.5-turbo-0125	10	0.787 (0.772 – 0.800)	0.808 (0.800 – 0.818)	0.939 (0.929 – 0.949)	0.704 (0.686 – 0.720)	0.989 (0.988 – 0.990)	0.973 (0.971 – 0.975)
LLM	Llama-3.1-70B-Instruct	0	0.835 (0.823 – 0.849)	0.828 (0.818 – 0.840)	0.978 (0.967 – 0.986)	0.769 (0.751 – 0.787)	0.997 (0.997 – 0.998)	0.972 (0.970 – 0.974)
LLM	Llama-3.3-70B-Instruct	10	0.852 (0.828 – 0.872)	0.804 (0.776 – 0.826)	**0.992** **(0.984 – 0.998)**	0.793 (0.758 – 0.819)	0.996 (0.993 – 0.998)	0.966 (0.960 – 0.971)
LLM	DeepSeek-R1-Distill-Llama-70B	0	0.871 (0.857 – 0.883)	0.860 (0.853 – 0.869)	0.958 (0.948 – 0.970)	0.831 (0.814 – 0.847)	0.991 (0.990 – 0.992)	0.984 (0.983 – 0.985)
LLM	o3-mini-2025-01-31	5	0.883 (0.873 – 0.892)	0.911 (0.904 – 0.917)	0.899 (0.879 – 0.914)	0.893 (0.881 – 0.902)	0.986 (0.985 – 0.988)	**0.994** **(0.993 – 0.995)**
LLM	gpt-4o-2024-08-06	0	0.885 (0.874 – 0.897)	0.898 (0.891 – 0.908)	0.961 (0.950 – 0.970)	0.857 (0.837 – 0.875)	0.991 (0.991 – 0.992)	0.989 (0.988 – 0.990)
LLM	Llama-DrugDetector-8B	3	0.909 (0.894 – 0.922)	**0.939** **(0.934 – 0.944)**	0.909 (0.891 – 0.924)	**0.914** **(0.900 – 0.928)**	0.992 (0.991 – 0.993)	**0.994** **(0.993 – 0.995)**
LLM	Llama-DrugDetector-70B	0	**0.917** **(0.905 – 0.928)**	0.926 (0.920 – 0.932)	0.959 (0.947 – 0.968)	0.894 (0.882 – 0.907)	0.994 (0.993 – 0.995)	0.993 (0.992 – 0.994)

**Table 6:** Mean F1-Scores on the held-out test set (n=6443) with bootstrapped lower and upper bounds for each detector and drug class. Abbreviations: Methamphetamine (Meth.), Benzodiazepine (Benzo.), Prescription Opioid Misuse (Rx. Opioids), Injection Drug Use (IDU). Overall performance, requiring simultaneous correct classification across all classes, is also reported.

Detector	Shots	Heroin	Cocaine	Meth.	Benzos.	Rx. Opioids	Cannabis	IDU	Any	Overall
biobert-v1.1	0	0.771 (0.758 – 0.787)	0.480 (0.476 – 0.484)	0.497 (0.496 – 0.498)	0.490 (0.489 – 0.491)	0.495 (0.494 – 0.496)	0.502 (0.494 – 0.513)	0.763 (0.752 – 0.773)	0.946 (0.941 – 0.951)	0.276 (0.271 – 0.281)
Bio_ClinicalBERT	0	0.801 (0.790 – 0.815)	0.478 (0.476 – 0.480)	0.497 (0.496 – 0.498)	0.490 (0.488 – 0.491)	0.495 (0.494 – 0.495)	0.495 (0.494 – 0.495)	0.775 (0.763 – 0.788)	0.939 (0.934 – 0.944)	0.279 (0.276 – 0.283)
bert-base-uncased	0	0.822 (0.809 – 0.837)	0.482 (0.477 – 0.488)	0.497 (0.497 – 0.498)	0.490 (0.489 – 0.491)	0.518 (0.495 – 0.549)	0.510 (0.495 – 0.535)	0.765 (0.753 – 0.776)	0.952 (0.946 – 0.958)	0.295 (0.285 – 0.305)
ClinicalBERT	0	0.844 (0.833 – 0.855)	0.477 (0.475 – 0.479)	0.496 (0.495 – 0.497)	0.494 (0.489 – 0.503)	0.495 (0.494 – 0.495)	0.573 (0.543 – 0.604)	0.755 (0.745 – 0.767)	0.944 (0.939 – 0.949)	0.308 (0.298 – 0.318)
MedLlama3-8B	0	0.167 (0.159 – 0.177)	0.680 (0.665 – 0.693)	0.502 (0.492 – 0.513)	0.899 (0.879 – 0.919)	0.658 (0.629 – 0.682)	0.453 (0.444 – 0.462)	0.642 (0.631 – 0.654)	0.414 (0.403 – 0.424)	0.403 (0.392 – 0.413)
Llama3-Med42-70B	0	0.767 (0.755 – 0.781)	0.869 (0.857 – 0.881)	0.732 (0.693 – 0.767)	0.834 (0.814 – 0.854)	0.613 (0.588 – 0.633)	0.725 (0.694 – 0.748)	0.759 (0.747 – 0.768)	0.932 (0.927 – 0.937)	0.608 (0.591 – 0.626)
Llama3-Med42-8B	10	0.731 (0.716 – 0.746)	0.879 (0.869 – 0.891)	0.702 (0.667 – 0.730)	0.802 (0.783 – 0.824)	0.620 (0.598 – 0.642)	0.762 (0.729 – 0.793)	0.894 (0.886 – 0.905)	0.937 (0.931 – 0.943)	0.624 (0.605 – 0.644)
Llama3-OpenBioLLM-8B	0	0.907 (0.898 – 0.918)	0.955 (0.945 – 0.965)	0.780 (0.742 – 0.817)	0.793 (0.758 – 0.823)	0.648 (0.621 – 0.690)	0.852 (0.821 – 0.889)	0.868 (0.858 – 0.879)	0.760 (0.748 – 0.769)	0.677 (0.657 – 0.695)
Llama-3.1-8B-Instruct	0	0.874 (0.861 – 0.884)	0.959 (0.953 – 0.966)	0.739 (0.700 – 0.775)	0.843 (0.826 – 0.866)	0.575 (0.557 – 0.592)	0.904 (0.881 – 0.927)	0.890 (0.880 – 0.899)	0.907 (0.900 – 0.915)	0.706 (0.691 – 0.722)
DeepSeek-R1-Distill-Llama-8B	5	0.880 (0.868 – 0.893)	0.935 (0.923 – 0.944)	0.806 (0.765 – 0.848)	0.817 (0.790 – 0.840)	0.705 (0.666 – 0.737)	0.859 (0.832 – 0.885)	0.883 (0.875 – 0.893)	0.853 (0.843 – 0.861)	0.716 (0.694 – 0.736)
Llama3-OpenBioLLM-70B	0	0.831 (0.819 – 0.843)	0.979 (0.973 – 0.986)	0.849 (0.805 – 0.881)	0.891 (0.871 – 0.908)	0.662 (0.637 – 0.688)	0.919 (0.893 – 0.944)	0.929 (0.919 – 0.936)	0.887 (0.880 – 0.894)	0.769 (0.747 – 0.789)
gpt-3.5-turbo-0125	10	0.880 (0.868 – 0.893)	0.973 (0.966 – 0.979)	0.806 (0.769 – 0.843)	0.876 (0.858 – 0.897)	0.714 (0.688 – 0.740)	0.950 (0.928 – 0.973)	0.926 (0.917 – 0.933)	0.949 (0.944 – 0.954)	0.787 (0.772 – 0.800)
Llama-3.1-70B-Instruct	0	0.895 (0.884 – 0.905)	0.992 (0.988 – 0.995)	0.932 (0.902 – 0.955)	0.928 (0.915 – 0.941)	0.655 (0.636 – 0.680)	0.973 (0.959 – 0.988)	0.928 (0.920 – 0.936)	0.976 (0.973 – 0.980)	0.835 (0.823 – 0.849)
Llama-3.3-70B-Instruct	10	0.858 (0.827 – 0.881)	0.983 (0.968 – 0.994)	**1.000** **(1.000 – 1.000)**	0.969 (0.941 – 0.993)	0.655 (0.598 – 0.708)	0.975 (0.928 – 1.000)	0.925 (0.906 – 0.946)	0.965 (0.953 – 0.976)	0.852 (0.828 – 0.872)
DeepSeek-R1-Distill-Llama-70B	0	0.891 (0.880 – 0.902)	0.994 (0.990 – 0.996)	0.943 (0.910 – 0.973)	0.952 (0.938 – 0.964)	0.729 (0.699 – 0.759)	0.989 (0.979 – 0.998)	0.976 (0.971 – 0.981)	0.961 (0.956 – 0.967)	0.871 (0.857 – 0.883)
o3-mini-2025-01-31	5	**0.985** **(0.980 – 0.990)**	0.993 (0.990 – 0.996)	0.975 (0.961 – 0.989)	0.888 (0.864 – 0.910)	0.726 (0.697 – 0.753)	0.983 (0.966 – 0.994)	**0.993** **(0.990 – 0.996)**	0.948 (0.942 – 0.954)	0.883 (0.873 – 0.892)
gpt-4o-2024-08-06	0	0.965 (0.958 – 0.971)	0.988 (0.983 – 0.993)	0.962 (0.935 – 0.981)	**0.971** **(0.961 – 0.980)**	0.690 (0.662 – 0.714)	0.975 (0.959 – 0.988)	0.987 (0.983 – 0.991)	0.965 (0.961 – 0.969)	0.885 (0.874 – 0.897)
Llama-DrugDetector-8B	3	0.981 (0.977 – 0.986)	0.991 (0.986 – 0.995)	0.948 (0.919 – 0.972)	0.932 (0.912 – 0.948)	**0.830** **(0.791 – 0.868)**	0.977 (0.961 – 0.989)	0.983 (0.979 – 0.987)	0.966 (0.961 – 0.970)	0.909 (0.894 – 0.922)
Llama-DrugDetector-70B	0	0.943 (0.935 – 0.951)	**0.994** **(0.991 – 0.997)**	0.964 (0.940 – 0.982)	0.969 (0.957 – 0.981)	0.815 (0.784 – 0.848)	**0.989** **(0.981 – 0.996)**	0.990 (0.987 – 0.993)	**0.976** **(0.973 – 0.981)**	**0.917** **(0.905 – 0.928)**

**Table 7:** Summary of common errors made by the best performing detector — our fine-tuned DrugDetector-70B. Percentages are calculated relative to the full test set and may slightly overstate error rates, as multiple issues can occur within a single instance.

Error type	Error description	Count	Percent
Insufficient Evidence	Assuming IDVU implied heroin when never explicitly mentioned	126	1.96%
	Assuming illicit use of prescription opioids without evidence	103	1.60%
	Assuming illicit use of benzodiazepines without evidence	9	0.14%
	Assuming specific substance mentioned in vague sentence	8	0.12%
	Assuming opiate/opioid use means heroin use	7	0.11%
	Assuming HepC means IVDU when never explicitly mentioned	5	0.08%
Missed Evidence	Simple failures to identify substance	113	1.75%
	Missed negation of drug use (e.g. patient denied use)	11	0.17%
	Missed illicit nature of use (e.g. prescription opioids)	2	0.03%
Confusion	Hallucinated drug use where none existed	30	0.47%
	Confusing family substance use for patient use	4	0.06%
	Confusing medical recommendation for actual use	3	0.05%
	Confusing typos (e.g. Klopopins → Klonopin)	2	0.03%
	**Total**	**433**	**6.72%**

## Data Availability

The DrugDetection dataset supporting this study is publicly available on PhysioNet (https://physionet.org/content/mimic-ext-drug-detection/1.0.0/) under the name MIMIC-Ext-DrugDetection Harel-Canada et al. (2025). As this dataset is derived from MIMIC data, access requires investigators to be credentialed PhysioNet users and adhere to the MIMIC Data Use Agreement, which includes completing the CITI “Data or Specimens Only Research” training.

## Footnotes

1 To further control for class imbalance, we normalize precision, recall, and F1 with *macro-averaging* This calculates metrics for each label via their *unweighted* means, effectively ignoring overall support for each class. This results in *lowering scores* relative to alternative normalizations like micro- or weighted-averaging, which yield substantially higher scores due to stronger performance of negative classes. For more details, see: https://scikit-learn.org/stable/modules/generated/sklearn.metrics.f1_score.html.

2 We fine-tune DeepSeek-R1-Distill-Llama (8B and 70B) based on the best performance observed across both zero- and few-shot testing. The performance of DeepSeek-R1-Distill-Llama-8B was significantly improved in the few-shot setting (F1: 0.716 at 5-shot vs F1: 0.706 for Llama-3.1–8B-Instruct’s best).

3 Recall that “Overall” reflects the correct identification of all substances present in the medical text simultaneously — i.e., a single misclassified drug nullifies otherwise correct multi-label predictions. This explains the divergence between peak single-substance F1 (0.994) and overall performance (0.917) for Llama-DrugDetector-70B.

4
https://huggingface.co/fabriceyhc/Llama-DrugDetector-70B

## Appendix A. Supplementary data

Supplementary material related to this article can be found online at https://doi.org/10.1016/j.drugalcdep.2025.112888.
