# Supplementary material for: Enhancing substance use detection in clinical notes with large language models
Source: Drug Alcohol Depend. Author manuscript; Available in PMC 2026 Jun 13. (PMC13263133; doi:10.1016/j.drugalcdep.2025.112888)
Supplement: 1 [file NIHMS2183821-supplement-1.pdf]

to surveil complex and emerging patterns of polysubstance use in the united states, International Journal of Drug Policy (2024) 104397.

[59] Z. Zhou, X. Ning, K. Hong, T. Fu, J. Xu, S. Li, Y. Lou, L. Wang, Z. Yuan, X. Li, S. Yan, G. Dai, X.-P. Zhang, Y. Dong, Y. Wang, A survey on efficient inference for large language models (2024). arXiv:2404.14294.
URL https://arxiv.org/abs/2404.14294

[60] OpenAI, Openai o3-mini, https://openai.com/index/openai-o3-mini/, [Accessed 04-16-2025] (2024).

[61] L. Tunstall, N. Reimers, U. E. S. Jo, L. Bates, D. Korat, M. Wasserblat, O. Pereg, Efficient few-shot learning without prompts (2022). doi:10.48550/ARXIV.2209.11055.
URL https://arxiv.org/abs/2209.11055

[62] Y. Zheng, R. Zhang, J. Zhang, Y. Ye, Z. Luo, Z. Feng, Y. Ma, Llamafactory: Unified efficient fine-tuning of 100+ language models (2024).
URL http://arxiv.org/abs/2403.13372

[63] E. J. Hu, Y. Shen, P. Wallis, Z. Allen-Zhu, Y. Li, S. Wang, W. Chen, Lora: Low-rank adaptation of large language models, CoRR abs/2106.09685 (2021). arXiv:2106.09685.
URL https://arxiv.org/abs/2106.09685

[64] I. Loshchilov, F. Hutter, Fixing weight decay regularization in adam, CoRR abs/1711.05101 (2017). arXiv:1711.05101.
URL http://arxiv.org/abs/1711.05101

Appendix A. Annotation Guide

We provide detailed guidelines and clarifications for annotators involved in the study. The section begins with the Substance Annotation Guide in 2, which outlines the specific instructions for categorizing and recording mentions of substance use in patient chart notes. Following the table, a series of clarifications address common scenarios and provide precise rules for annotators to follow. This comprehensive guide ensures consistency and accuracy across all annotations.

Drug use (dichotomous)	Yes	There is a mention that the case has used illicit drugs or has a history of illicit drug use. Illicit substances would include cannabis/marijuana, cocaine, crack, amphetamines/methamphetamine, opioids (heroin, prescription opioids, fentanyl, methadone), hallucinogens, benzodiazepines, inhalants. Use of tobacco or alcohol alone should be labeled as “no”. Use of prescribed drugs as indicated like opioids or benzodiazepines, should be labeled as “no”. Other broad terms include OUD, opioid use disorder, addict, drug addict, drug dependent, polysubstance use, injection illicit drug use, injection ____ [specify drug], [specify drug] injector, IVDU, IVDA, PWID, IDU, intravenous drug user, intravenous drug abuser, injection drug user, skin popping, muscle popping. Key areas to look are in the History of Present Illness (HPI), social history, and assessment and plan.
	No	There is no mention that the case used drugs.
Injection drug use (dichotomous)	Yes	There is a mention that the case injected drugs or has a history of injection drug use. Terms may include: injection illicit drug use, injection ____ [specify drug], [specify drug] injector, IVDU, IVDA, PWID, IDU, intravenous drug user, intravenous drug abuser, injection drug user, skin popping, muscle popping. Key areas to look are in the History of Present Illness (HPI), social history, physical exam, and assessment and plan.
	No	There is no mention that the case injected drugs.
Type (categorical, multiple selection)	Heroin	Specified use of “heroin”. Other terms include “black tar”, “powder heroin”, “china white”, “dope”, “smack”, “horse”.
	Prescription opioid	Specified illicit use of a prescription opioid. Examples include oxycodone, oxymorphone, hydrocodone, hydromorphone, Dilaudid, Opana, Norco, Percocet, Vicodin, oxys, methadone, buprenorphine. Fentanyl use should be classified with its own label.
	Fentanyl	Specified injection, smoking or snorting of “fentanyl”.
	Cocaine / crack	Specified injection, smoking, or snorting of “cocaine”, “crack” or “crack cocaine”.
	Methamphetamine / amphetamine	Specified injection, smoking, or snorting of methamphetamine or amphetamines. Example terms include “meth”, “methamphetamine”, “amphetamine”, “speed”, “ice”, “crystal”. Notes may specify that the case used methamphetamine.
	Benzodiazepines	Specified illicit use of benzos, benzodiazepines, diazepam, valium, alprazolam, zanax, clonazepam, konipin.
	Cannabis / marijuana	Specified use of cannabis, marijuana, THC, pot.
	Not specified	Note does not specify drug that case injected. Examples may state that the participants is an [IDU, IVDU, IVDA, PWID, polysubstance user] but did not state what drug was injected/used.

Figure 2: Substance Annotation Guidelines. Note: This guide specifies the verbatim terms, including potentially stigmatizing language (e.g., ‘addict’, ‘abuser’), that annotators were trained to recognize in the source clinical notes. This was a procedural necessity for accurately labeling the data. The authors have used person-first language in all descriptive and narrative sections of this paper.

Annotator clarifications:

1. Any mention of injection drug use (IDU or IVDU) will be marked as such and also as drug use – yes
2. Annotations must be within a single sentence
3. Annotate for every substance that is mentioned (if multiple are mentioned)
4. If there is a drug name mentioned, check to see which drug class it falls under, and annotate with the respected drug class.
5. "not specified" will account for notes that mention: "polysubstance abuse", "substance abuse", "using drugs", mentions "opiates" broadly, etc.
   (a) Disclaimer: IVDU is NOT marked as "not specified"
6. Chart notes that indicate patient denies or does not take drugs will be marked as drug use no, and the corresponding substances mentioned will not be annotated.
   (a) Ex: 'patient denies using heroin'
      i. Annotation: Drug use no. No mark for heroin.
   (b) Ex: 'does not use heroin or cocaine'
      i. Annotation: Drug use no. No mark for heroin or cocaine.
7. If the chart mentioned "no IDU", there will also be an annotation for "drug use no"
8. Make sure all drug use that is being annotated is illicit drug use, and not prescribed medication.
9. For unclear opioid examples in which the medical note mentions "opiate overdose" or "opioid withdrawal", mark as "not specified"
   (a) Only mark as prescription opioid if it explicitly says so

Appendix B. Model Descriptions

We investigate a range of language models, categorized into two main classes: BERT-style encoders and GPT-style decoders. These models are further explored across different dimensions, including base models, models with generic medical pre-training, and models with fine-tuning.

BERT-Style Encoders

BERT-style encoders are designed to convert input text into rich, contextualized representations. They excel in tasks that require a deep understanding of the text, such as text classification. We selected models from this category due to their proven effectiveness in capturing the meaning and relationships within medical and clinical texts.

1. **BERT** [17]: The standard BERT model, trained on a large corpus of general English text, serving as a baseline for comparison. Model source: `https://huggingface.co/google-bert/bert-base-uncased`.
2. **BioBERT** [18]: A variant of BERT pre-trained on Pubmed datasets containing 18 billion words from biomedical literature and has demonstrated an improved ability to process biomedical text. Model source: `https://huggingface.co/dmis-lab/biobert-v1.1`.
3. **ClinicalBERT** [20]: A variant of BERT pre-trained pre-trained on clinical text (MIMIC III) [30], providing specialized adaptation to clinical language. Model source: `https://huggingface.co/medicalai/ClinicalBERT`.
4. **Bio_ClinicalBERT** [19]: An extension of BioBERT with additional training on clinical notes from MIMIC III, enhancing its ability to handle clinical language and terminologies. Model source: `https://huggingface.co/emilyalsentzer/Bio_ClinicalBERT`

GPT-Style Decoders

In contrast, GPT-style decoders are designed for text generation, predicting the next word in a sequence based on the preceding context. We adapt these decoders for classification tasks by prompting them to generate responses that mimic classification outputs. We study a mix of proprietary LLMs – `GPT-3.5-Turbo` [21], `GPT-4-Turbo` [22], and `GPT-4o` [36] – as well as open-source alternatives. Among the open-source LLMs, we examine `DeepSeek-R1-Distill-Llama` [37] and the Llama-3 series of models [23],

including `Llama-3-Instruct` and its updated variant `Llama-3.1-Instruct`, which offers strong performance across diverse tasks. The open-source LLMs offer comparable performance to most proprietary LLMs and have the distinct advantage of being hosted locally, which is ideal for medical applications where privacy requirements complicate sharing medical data with third-party AI vendors.

1. **gpt-3.5-turbo-0125** [21]: An advanced generative model known for its robust language understanding and generation capabilities.
2. **gpt-4o** [36]: A multimodal variant of GPT-4 [22], optimized for specific applications, providing enhanced capabilities in understanding and generating complex texts.
3. **o3-mini** [60]: A reasoning model optimized for STEM tasks like math, coding, and science, offering adjustable reasoning levels and faster response times compared to its predecessors.
4. **Meta-Llama-3-Instruct** [23]: A series of instruction-following models with different parameter sizes (8B, 70B) designed for general purposes, providing robust performance across various tasks.
   - `https://huggingface.co/meta-llama/Meta-Llama-3-8B-Instruct`
   - `https://huggingface.co/meta-llama/Meta-Llama-3-70B-Instruct`
5. **Meta-Llama-3.1-Instruct** [23]: An updated series of the Meta-Llama-3 models supporting longer inputs, improved multilingualism, and other improvements in three parameter sizes (8B, 70B, 405B).
   - `https://huggingface.co/meta-llama/Meta-Llama-3.1-8B-Instruct`
   - `https://huggingface.co/meta-llama/Meta-Llama-3.1-70B-Instruct`
   - `https://huggingface.co/meta-llama/Meta-Llama-3.1-405B-Instruct` (Not included in study)
6. **Meta-Llama-3.3-Instruct** [23]: A more efficient and powerful version of the 70B model of the Llama-3 family, generally regarded as being on par with `Meta-Llama-3.1-405B-Instruct` at a fraction of the size.
   - `https://huggingface.co/meta-llama/Llama-3.3-70B-Instruct`
7. **DeepSeek-R1-Distill-Llama** [37]: A fine-tuned version of Llama-3.1 specifically optimized for mathematical, scientific, and logical reasoning capabilities.
   - `https://huggingface.co/deepseek-ai/DeepSeek-R1-Distill-Llama-8B`

- https://huggingface.co/deepseek-ai/DeepSeek-R1-Distill-Llama-70B

8. **MedLlama3** [38]: The model is a fine-tuned version of `Llama-3-8B` using publicly available medical data.
    - https://huggingface.co/ProbeMedicalYonseiMAILab/medllama3-v20[6]
9. **Llama3-Med42** [40]: A collection of open-access clinical LLMs developed by M42, designed to enhance accessibility to medical knowledge. Based on Llama-3 and available with either 8 or 70 billion parameters, these generative AI systems deliver high-quality responses to medical questions.
    - https://huggingface.co/m42-health/Llama3-Med42-8B
    - https://huggingface.co/m42-health/Llama3-Med42-70B
10. **Llama3-OpenBioLLM** [39]: Similar to Llama3-Med42, these models are developed by Saama AI Labs and pre-trained on a diverse set of biomedical literature to support a wide range of biomedical NLP applications.
    - https://huggingface.co/aaditya/Llama3-OpenBioLLM-8B
    - https://huggingface.co/aaditya/Llama3-OpenBioLLM-70B
11. **Llama-DrugDetector**: Our few-shot fine-tuned version of DeepSeek-R1-Distill-Llama optimized for drug detection tasks.
    - https://huggingface.co/fabriceyhc/Llama-DrugDetector-8B
    - https://huggingface.co/fabriceyhc/Llama-DrugDetector-70B

[6]As of April 17, 2025, the model appears to have been removed from Huggingface for unknown reasons.

Appendix C. Training

To better understand the degree to which "off-the-shelf" models can be improved with few-shot training, we employed a comprehensive fine-tuning process on both BERT-style models and GPT-style LLMs.

We fine-tuned all four BERT-style models – BERT, BioBERT, ClinicalBERT, and Bio_ClinicalBERT – using our DrugDetection dataset. Since these models are relatively small (less than 1B parameters), they train in less than 2 hours each on a single Nvidia A6000 48GB GPU. For the fine-tuning process, we utilized SetFit [61] with a multi-output target strategy to facilitate multi-label training. The input to the models consisted solely of the medical note, while the output is an 8-dimensional binary vector, indicating the presence or absence of each drug class within the note. The models were trained for up to 20 epochs, with an early stopping criterion if the validation loss did not decrease after 10 attempts. On average, the models achieved convergence within 1.65 epochs.

Given the resource-intensive and time-consuming nature of training LLMs, we limited our focus to the Deepseek-R1-Distill-Llama models with 8 billion (8B) and 70 billion (70B) parameters. We used llama-factory [62] scripts for supervised fine-tuning with Low-Rank Adaptation (LoRA) [63] to train these models on the DrugDetection dataset. The training process for both models spanned 3 epochs, without early stopping, as this feature is not currently supported in the utilized framework. Fine-tuning the 8B model required approximately 8 hours on 2 Nvidia A6000 48GB GPUs, while the 70B required approximately 24hrs on 8 GPUs of the same type.

Standard configurations for supervised fine-tuning were applied, including using an AdamW optimizer [64] with a weight decay of 0.01 and a learning rate of 0.0003. Additionally, a 100-step cosine warmup schedule was implemented. Cross-entropy loss was computed between the expected labels and the generated outputs to guide the optimization process.

Appendix D. Prompt Template

After extensive iterations, we developed an effective prompt template. Our approach involved prototyping a template, testing it on a small development set (separate from the DrugDetection dataset), and analyzing the errors made by the LLMs. These error analyses provided actionable insights, enabling us to add "guard rails" in the special notes section. These adjustments significantly enhanced performance for certain classes, especially for detecting prescription opioid misuse (i.e. initially around ~60% F1 accuracy, up to ~95%). Depending on the LLM, we would either use `guidance` [42] or `langchain` [43] with JSON output parsing to prompt for `True` or `False` labels for each drug in an extractable format.

Listing 1: Zero-shot drug detection prompt featuring task and drug descriptions with special notes curated from several rounds of error analysis.

```
You are a medical expert medical expert conducting important research where accuracy is
    essential.

Task: Analyze the provided medical text for references to illicit drug use, focusing on
    specific drug categories and adhering to special considerations.

Drug Categories to Identify:
  - Heroin: Heroin is an illegal opioid drug known for its high potential for
      addiction and overdose.
  - Cocaine: Cocaine is a powerful stimulant drug that is often used for its euphoric
      effects.
  - Methamphetamine: Methamphetamine (including illicit amphetamine use, but not
      prescribed amphetamines for ADHD) is a potent central nervous system stimulant
      that is highly addictive.
  - Benzodiazepine: Benzodiazepines (only if being misused or used illicitly, not if
      taken as prescribed) are a class of psychoactive drugs commonly prescribed for
      anxiety, insomnia, and other conditions but can be used for their sedative
      effects.
  - Prescription Opioids: Prescription opioids (only if being misused or used
      illicitly, not if taken as prescribed) are medications typically prescribed for
      pain relief but can be highly addictive when misused.
  - Cannabis: Cannabis, also known as marijuana, is often used recreationally or
      medicinally but can be illegal depending on the jurisdiction.
  - Injection Drugs: Injection drug use (IDU, IVDA, IVDU) refers to the use of
      illicit drugs administered via needles, often associated with higher risks of
      infectious diseases.
  - General Drugs: General drug use refers to the use of any illegal or illicit
      substances.

Special Considerations:
1. For unspecified "substance dependence," mark only "General Drug Use" as True
2. If no substances or drug-related behaviors are mentioned, mark all categories as
    False
3. Mark as False if information is missing or not explicitly stated
4. Warnings against drug use do not indicate patient use
5. Medical recommendations about drugs do not indicate patient use
6. Family drug use is not relevant to the patient
7. Only identify inappropriate use of opioids and benzodiazepines (e.g., acquired from
    friends or streets)
8. Patient denial of drug use should be marked as False

### The medical text to evaluate:
{medical_text}
```

Appendix E. Detailed Model Performance By Drug Class

We provide detailed breakdowns of detector performance by drug class, with each table focusing on a specific metric. For instance, Table 8 shows accuracy. Detectors are sorted by "Overall" performance, which reflects exact matches across all classes simultaneously. This "Overall" metric is often lower due to the complexity added by the frequent co-occurrence of multiple drug classes in medical notes.

Detector	Shots	Heroin	Cocaine	Meth.	Benzo.	Rx. Opioids	Cannabis	IDU	Any	Overall
MedLlama3-v20-8B	3	0.344<br>(0.335 - 0.356)	0.845<br>(0.834 - 0.855)	0.916<br>(0.909 - 0.922)	0.922<br>(0.916 - 0.928)	0.818<br>(0.809 - 0.826)	0.845<br>(0.837 - 0.853)	0.475<br>(0.462 - 0.489)	0.589<br>(0.578 - 0.601)	0.198<br>(0.190 - 0.209)
Llama3-Med42-70B	0	0.859<br>(0.850 - 0.867)	0.950<br>(0.946 - 0.955)	0.976<br>(0.972 - 0.979)	0.967<br>(0.963 - 0.971)	0.905<br>(0.899 - 0.912)	0.958<br>(0.954 - 0.962)	0.820<br>(0.810 - 0.828)	0.932<br>(0.926 - 0.938)	0.661<br>(0.646 - 0.672)
biobert-v1.1	0	0.893<br>(0.886 - 0.901)	0.915<br>(0.908 - 0.922)	0.988<br>(0.985 - 0.990)	0.961<br>(0.957 - 0.966)	0.980<br>(0.977 - 0.983)	0.980<br>(0.977 - 0.983)	0.827<br>(0.818 - 0.835)	0.946<br>(0.940 - 0.950)	0.666<br>(0.653 - 0.679)
ClinicalBERT	0	0.925<br>(0.919 - 0.931)	0.913<br>(0.906 - 0.919)	0.985<br>(0.982 - 0.987)	0.962<br>(0.958 - 0.967)	0.979<br>(0.976 - 0.982)	0.975<br>(0.972 - 0.979)	0.826<br>(0.818 - 0.832)	0.944<br>(0.940 - 0.949)	0.684<br>(0.672 - 0.693)
Bio_ClinicalBERT	0	0.914<br>(0.906 - 0.921)	0.915<br>(0.909 - 0.922)	0.988<br>(0.985 - 0.991)	0.959<br>(0.955 - 0.964)	0.979<br>(0.975 - 0.982)	0.978<br>(0.974 - 0.982)	0.853<br>(0.844 - 0.862)	0.939<br>(0.932 - 0.944)	0.689<br>(0.676 - 0.699)
bert-base-uncased	0	0.929<br>(0.923 - 0.934)	0.917<br>(0.912 - 0.922)	0.989<br>(0.986 - 0.991)	0.961<br>(0.957 - 0.965)	0.979<br>(0.975 - 0.982)	0.980<br>(0.977 - 0.983)	0.830<br>(0.820 - 0.839)	0.952<br>(0.947 - 0.958)	0.691<br>(0.682 - 0.701)
Llama3-OpenBioLLM-8B	0	0.962<br>(0.958 - 0.966)	0.987<br>(0.984 - 0.989)	0.985<br>(0.982 - 0.987)	0.977<br>(0.974 - 0.980)	0.964<br>(0.960 - 0.968)	0.989<br>(0.986 - 0.991)	0.931<br>(0.925 - 0.936)	0.774<br>(0.764 - 0.783)	0.699<br>(0.690 - 0.708)
Llama3-Med42-8B	10	0.827<br>(0.816 - 0.838)	0.955<br>(0.950 - 0.960)	0.969<br>(0.964 - 0.975)	0.959<br>(0.953 - 0.964)	0.911<br>(0.904 - 0.917)	0.970<br>(0.965 - 0.975)	0.934<br>(0.928 - 0.939)	0.938<br>(0.932 - 0.942)	0.716<br>(0.705 - 0.727)
Llama-3.1-8B-Instruct	10	0.883<br>(0.875 - 0.891)	0.978<br>(0.975 - 0.981)	0.985<br>(0.982 - 0.988)	0.978<br>(0.975 - 0.982)	0.885<br>(0.877 - 0.892)	0.989<br>(0.986 - 0.991)	0.945<br>(0.939 - 0.950)	0.950<br>(0.947 - 0.955)	0.729<br>(0.720 - 0.740)
DeepSeek-R1-Distill-Llama-8B	10	0.919<br>(0.912 - 0.925)	0.978<br>(0.975 - 0.981)	0.985<br>(0.982 - 0.987)	0.977<br>(0.974 - 0.981)	0.968<br>(0.965 - 0.971)	0.987<br>(0.985 - 0.989)	0.938<br>(0.932 - 0.943)	0.892<br>(0.884 - 0.898)	0.762<br>(0.752 - 0.770)
Llama3-OpenBioLLM-70B	10	0.885<br>(0.877 - 0.894)	0.980<br>(0.977 - 0.983)	0.983<br>(0.981 - 0.986)	0.972<br>(0.968 - 0.976)	0.910<br>(0.903 - 0.917)	0.985<br>(0.982 - 0.988)	0.963<br>(0.959 - 0.968)	0.968<br>(0.964 - 0.972)	0.787<br>(0.775 - 0.796)
Llama-3.3-70B-Instruct	10	0.923<br>(0.906 - 0.941)	0.994<br>(0.989 - 0.999)	**1.000**<br>**(1.000 - 1.000)**	0.994<br>(0.988 - 0.999)	0.923<br>(0.904 - 0.940)	0.999<br>(0.996 - 1.000)	0.955<br>(0.944 - 0.967)	0.966<br>(0.953 - 0.977)	0.804<br>(0.776 - 0.826)
gpt-3.5-turbo-0125	10	0.942<br>(0.936 - 0.947)	0.991<br>(0.989 - 0.993)	0.987<br>(0.984 - 0.990)	0.978<br>(0.974 - 0.982)	0.962<br>(0.957 - 0.966)	0.996<br>(0.994 - 0.998)	0.957<br>(0.952 - 0.963)	0.948<br>(0.943 - 0.953)	0.808<br>(0.800 - 0.818)
Llama-3.1-70B-Instruct	0	0.949<br>(0.944 - 0.955)	0.997<br>(0.996 - 0.999)	0.997<br>(0.996 - 0.998)	0.989<br>(0.986 - 0.991)	0.930<br>(0.924 - 0.936)	0.998<br>(0.997 - 0.999)	0.957<br>(0.952 - 0.962)	0.976<br>(0.973 - 0.979)	0.828<br>(0.818 - 0.840)
DeepSeek-R1-Distill-Llama-70B	10	0.936<br>(0.931 - 0.942)	0.994<br>(0.992 - 0.996)	0.995<br>(0.993 - 0.997)	0.990<br>(0.988 - 0.992)	0.970<br>(0.966 - 0.975)	0.998<br>(0.996 - 0.999)	0.989<br>(0.986 - 0.991)	0.957<br>(0.953 - 0.961)	0.860<br>(0.853 - 0.869)
gpt-4o-2024-08-06	0	0.985<br>(0.982 - 0.988)	0.996<br>(0.995 - 0.998)	0.998<br>(0.997 - 0.999)	0.996<br>(0.994 - 0.997)	0.949<br>(0.943 - 0.955)	0.998<br>(0.997 - 0.999)	0.993<br>(0.990 - 0.994)	0.965<br>(0.960 - 0.969)	0.898<br>(0.891 - 0.908)
o3-mini-2025-01-31	10	0.994<br>(0.991 - 0.995)	0.998<br>(0.997 - 0.999)	0.999<br>(0.998 - 1.000)	0.987<br>(0.984 - 0.990)	0.968<br>(0.964 - 0.971)	0.999<br>(0.998 - 1.000)	**0.996**<br>**(0.994 - 0.997)**	0.953<br>(0.948 - 0.958)	0.911<br>(0.904 - 0.917)
Llama-DrugDetector-70B	0	0.975<br>(0.972 - 0.978)	0.998<br>(0.997 - 0.999)	0.998<br>(0.997 - 0.999)	**0.996**<br>**(0.994 - 0.997)**	0.980<br>(0.977 - 0.984)	**0.999**<br>**(0.999 - 1.000)**	0.995<br>(0.994 - 0.997)	**0.976**<br>**(0.973 - 0.980)**	0.926<br>(0.920 - 0.932)
Llama-DrugDetector-8B	0	**0.996**<br>**(0.994 - 0.997)**	**0.998**<br>**(0.997 - 0.999)**	0.997<br>(0.996 - 0.998)	0.989<br>(0.987 - 0.991)	**0.986**<br>**(0.983 - 0.989)**	0.998<br>(0.998 - 0.999)	0.993<br>(0.991 - 0.995)	0.975<br>(0.971 - 0.978)	**0.939**<br>**(0.934 - 0.944)**

Table 8: Mean accuracy on the held-out test set (n=6443) with bootstrapped confidence intervals for each detector and drug class. The "Overall" scores may be lower than those for individual classes, as they reflect exact matches across all classes simultaneously, a task made more challenging by the frequent co-occurrence of multiple drug classes in many medical notes. Abbreviations: Methamphetamine (Meth.), Benzodiazepine (Benzo.), Prescription Opioid Misuse (Rx. Opioids), Injection Drug Use (IDU).

Detector	Shots	Heroin	Cocaine	Meth.	Benzos.	Rx. Opioids	Cannabis	IDU	Any	Overall
Bio_ClinicalBERT	0	0.820 (0.807 - 0.832)	0.498 (0.498 - 0.499)	0.500 (0.499 - 0.500)	0.497 (0.497 - 0.498)	0.499 (0.498 - 0.499)	0.499 (0.498 - 0.499)	0.838 (0.826 - 0.850)	0.939 (0.934 - 0.945)	0.311 (0.307 - 0.315)
bert-base-uncased	0	0.817 (0.803 - 0.833)	0.501 (0.499 - 0.504)	0.500 (0.500 - 0.500)	0.499 (0.498 - 0.499)	0.512 (0.499 - 0.530)	0.507 (0.499 - 0.521)	0.872 (0.864 - 0.879)	0.952 (0.947 - 0.958)	0.328 (0.322 - 0.335)
biobert-v1.1	0	0.809 (0.795 - 0.824)	0.499 (0.498 - 0.502)	0.500 (0.499 - 0.500)	0.499 (0.498 - 0.499)	0.500 (0.499 - 0.500)	0.503 (0.499 - 0.509)	0.875 (0.867 - 0.882)	0.946 (0.942 - 0.951)	0.329 (0.324 - 0.334)
ClinicalBERT	0	0.913 (0.901 - 0.923)	0.497 (0.496 - 0.498)	0.498 (0.497 - 0.499)	0.501 (0.499 - 0.506)	0.499 (0.498 - 0.499)	0.558 (0.534 - 0.586)	0.858 (0.848 - 0.867)	0.944 (0.939 - 0.949)	0.363 (0.356 - 0.371)
Llama3-OpenBioLLM-8B	0	0.903 (0.891 - 0.915)	0.943 (0.929 - 0.957)	0.921 (0.878 - 0.960)	0.732 (0.696 - 0.761)	0.700 (0.660 - 0.744)	0.850 (0.815 - 0.888)	0.851 (0.840 - 0.863)	0.769 (0.759 - 0.776)	0.680 (0.659 - 0.700)
DeepSeek-R1-Distill-Llama-8B	10	0.940 (0.933 - 0.948)	0.964 (0.953 - 0.972)	0.947 (0.923 - 0.974)	0.859 (0.827 - 0.886)	0.784 (0.737 - 0.828)	0.907 (0.877 - 0.935)	0.899 (0.889 - 0.908)	0.890 (0.881 - 0.899)	0.829 (0.810 - 0.845)
o3-mini-2025-01-31	0	**0.989 (0.984 - 0.993)**	0.993 (0.987 - 0.997)	0.969 (0.937 - 0.993)	0.834 (0.801 - 0.855)	0.876 (0.836 - 0.913)	0.979 (0.962 - 0.993)	0.992 (0.988 - 0.996)	0.942 (0.936 - 0.947)	0.899 (0.884 - 0.911)
Llama-DrugDetector-8B	3	**0.989 (0.984 - 0.993)**	0.991 (0.984 - 0.996)	0.958 (0.925 - 0.986)	0.960 (0.944 - 0.978)	0.795 (0.752 - 0.836)	0.975 (0.954 - 0.991)	0.977 (0.971 - 0.983)	0.966 (0.961 - 0.970)	0.909 (0.891 - 0.924)
gpt-3.5-turbo-0125	10	0.948 (0.940 - 0.956)	0.987 (0.981 - 0.993)	0.979 (0.959 - 0.994)	0.968 (0.953 - 0.981)	0.886 (0.853 - 0.913)	0.975 (0.954 - 0.991)	0.956 (0.949 - 0.963)	0.948 (0.943 - 0.954)	0.939 (0.929 - 0.949)
MedLlama3-8B	5	0.626 (0.620 - 0.631)	0.914 (0.909 - 0.920)	0.945 (0.917 - 0.963)	0.935 (0.920 - 0.950)	0.856 (0.822 - 0.882)	0.893 (0.864 - 0.912)	0.669 (0.662 - 0.675)	0.592 (0.586 - 0.598)	0.955 (0.943 - 0.966)
DeepSeek-R1-Distill-Llama-70B	0	0.965 (0.960 - 0.970)	**0.995 (0.990 - 0.998)**	0.941 (0.905 - 0.977)	0.978 (0.962 - 0.989)	0.952 (0.934 - 0.968)	0.991 (0.976 - 1.000)	0.988 (0.984 - 0.991)	0.961 (0.955 - 0.966)	0.958 (0.948 - 0.970)
Llama-DrugDetector-70B	0	0.981 (0.977 - 0.985)	0.993 (0.988 - 0.997)	0.946 (0.913 - 0.974)	0.976 (0.960 - 0.988)	0.954 (0.926 - 0.976)	0.990 (0.977 - 1.000)	**0.993 (0.990 - 0.995)**	**0.976 (0.973 - 0.980)**	0.959 (0.947 - 0.968)
gpt-4o-2024-08-06	3	0.967 (0.960 - 0.973)	0.988 (0.980 - 0.994)	0.993 (0.979 - 0.999)	0.970 (0.956 - 0.982)	0.953 (0.934 - 0.967)	0.983 (0.965 - 0.997)	0.988 (0.984 - 0.991)	0.968 (0.962 - 0.972)	0.967 (0.957 - 0.975)
Llama-3.1-8B-Instruct	3	0.911 (0.903 - 0.917)	0.979 (0.973 - 0.985)	0.979 (0.962 - 0.987)	0.973 (0.963 - 0.982)	0.899 (0.874 - 0.918)	0.979 (0.965 - 0.991)	0.948 (0.943 - 0.953)	0.940 (0.934 - 0.946)	0.967 (0.958 - 0.975)
Llama3-Med42-8B	0	0.906 (0.901 - 0.912)	0.978 (0.974 - 0.980)	0.971 (0.957 - 0.981)	0.966 (0.958 - 0.976)	0.901 (0.887 - 0.914)	0.962 (0.942 - 0.979)	0.962 (0.957 - 0.967)	0.937 (0.932 - 0.943)	0.968 (0.961 - 0.975)
Llama-3.1-70B-Instruct	5	0.933 (0.926 - 0.938)	0.990 (0.985 - 0.995)	0.982 (0.959 - 0.998)	**0.991 (0.986 - 0.994)**	0.938 (0.926 - 0.950)	0.989 (0.976 - 0.998)	0.966 (0.962 - 0.970)	0.971 (0.968 - 0.975)	0.983 (0.975 - 0.989)
Llama3-Med42-70B	10	0.853 (0.847 - 0.860)	0.955 (0.950 - 0.959)	0.979 (0.977 - 0.982)	0.975 (0.969 - 0.979)	0.913 (0.897 - 0.928)	0.968 (0.954 - 0.980)	0.885 (0.880 - 0.892)	0.931 (0.925 - 0.937)	0.990 (0.985 - 0.995)
Llama3-OpenBioLLM-70B	5	0.923 (0.917 - 0.928)	0.986 (0.984 - 0.988)	0.989 (0.988 - 0.991)	0.977 (0.968 - 0.984)	0.936 (0.923 - 0.949)	0.982 (0.967 - 0.990)	0.973 (0.970 - 0.976)	0.969 (0.966 - 0.973)	0.991 (0.984 - 0.996)
Llama-3.3-70B-Instruct	10	0.956 (0.948 - 0.964)	0.991 (0.978 - 0.999)	**1.000 (1.000 - 1.000)**	0.987 (0.956 - 0.999)	**0.960 (0.951 - 0.968)**	**0.999 (0.998 - 1.000)**	0.973 (0.967 - 0.981)	0.965 (0.954 - 0.976)	**0.992 (0.984 - 0.998)**

Table 9: Mean Sensitivity (recall) on the held-out test set (n=6443) with bootstrapped lower and upper bounds for each detector and drug class. Abbreviations: Methamphetamine (Meth.), Benzodiazepine (Benzo.), Prescription Opioid Misuse (Rx. Opioids), Injection Drug Use (IDU).

Detector	Shots	Heroin	Cocaine	Meth.	Benzos.	Rx. Opioids	Cannabis	IDU	Any	Overall
Bio_ClinicalBERT	0	0.786 (0.772 - 0.803)	0.459 (0.456 - 0.463)	0.494 (0.493 - 0.496)	0.482 (0.480 - 0.484)	0.491 (0.489 - 0.492)	0.491 (0.489 - 0.492)	0.746 (0.733 - 0.758)	0.940 (0.935 - 0.945)	0.256 (0.252 - 0.261)
biobert-v1.1	0	0.745 (0.731 - 0.761)	0.486 (0.456 - 0.551)	0.494 (0.493 - 0.496)	0.482 (0.480 - 0.484)	0.490 (0.489 - 0.492)	0.559 (0.489 - 0.690)	0.735 (0.724 - 0.744)	0.947 (0.943 - 0.952)	0.265 (0.240 - 0.300)
ClinicalBERT	0	0.802 (0.790 - 0.814)	0.459 (0.455 - 0.462)	0.494 (0.493 - 0.496)	0.517 (0.480 - 0.578)	0.490 (0.489 - 0.492)	0.601 (0.558 - 0.653)	0.728 (0.718 - 0.737)	0.944 (0.940 - 0.949)	0.288 (0.269 - 0.309)
MedLlama3-8B	0	0.561 (0.558 - 0.566)	0.653 (0.642 - 0.663)	0.528 (0.523 - 0.534)	0.903 (0.881 - 0.927)	0.610 (0.589 - 0.630)	0.527 (0.523 - 0.531)	0.667 (0.659 - 0.677)	0.719 (0.702 - 0.734)	0.304 (0.293 - 0.314)
bert-base-uncased	0	0.828 (0.810 - 0.843)	0.551 (0.459 - 0.661)	0.494 (0.493 - 0.495)	0.482 (0.480 - 0.484)	0.580 (0.491 - 0.683)	0.586 (0.490 - 0.733)	0.736 (0.725 - 0.746)	0.952 (0.948 - 0.958)	0.334 (0.290 - 0.378)
Llama3-Med42-70B	0	0.725 (0.714 - 0.738)	0.810 (0.797 - 0.825)	0.656 (0.624 - 0.687)	0.762 (0.739 - 0.784)	0.580 (0.565 - 0.593)	0.655 (0.631 - 0.674)	0.734 (0.725 - 0.743)	0.935 (0.930 - 0.940)	0.468 (0.451 - 0.485)
Llama3-Med42-8B	10	0.700 (0.687 - 0.711)	0.824 (0.813 - 0.839)	0.634 (0.607 - 0.656)	0.732 (0.713 - 0.755)	0.585 (0.570 - 0.599)	0.688 (0.659 - 0.717)	0.857 (0.847 - 0.871)	0.940 (0.934 - 0.945)	0.502 (0.483 - 0.522)
Llama-3.1-8B-Instruct	0	0.824 (0.809 - 0.837)	0.932 (0.920 - 0.942)	0.664 (0.631 - 0.697)	0.784 (0.767 - 0.810)	0.561 (0.550 - 0.571)	0.846 (0.813 - 0.877)	0.856 (0.845 - 0.866)	0.921 (0.915 - 0.928)	0.618 (0.602 - 0.637)
Llama3-OpenBioLLM-70B	0	0.780 (0.767 - 0.792)	0.963 (0.953 - 0.976)	0.773 (0.725 - 0.815)	0.828 (0.803 - 0.851)	0.610 (0.592 - 0.629)	0.873 (0.835 - 0.913)	0.897 (0.884 - 0.906)	0.904 (0.899 - 0.911)	0.659 (0.630 - 0.688)
DeepSeek-R1-Distill-Llama-8B	5	0.836 (0.821 - 0.851)	0.911 (0.894 - 0.925)	0.737 (0.694 - 0.776)	0.821 (0.789 - 0.852)	0.666 (0.631 - 0.699)	0.822 (0.793 - 0.850)	0.878 (0.866 - 0.890)	0.873 (0.865 - 0.881)	0.670 (0.639 - 0.696)
gpt-3.5-turbo-0125	10	0.836 (0.822 - 0.853)	0.959 (0.950 - 0.967)	0.728 (0.689 - 0.769)	0.816 (0.794 - 0.844)	0.653 (0.630 - 0.676)	0.927 (0.897 - 0.963)	0.902 (0.890 - 0.912)	0.949 (0.945 - 0.955)	0.704 (0.686 - 0.720)
Llama-3.1-70B-Instruct	0	0.848 (0.835 - 0.861)	0.989 (0.985 - 0.994)	0.912 (0.871 - 0.946)	0.882 (0.863 - 0.901)	0.605 (0.592 - 0.624)	0.958 (0.936 - 0.981)	0.895 (0.884 - 0.906)	0.976 (0.973 - 0.980)	0.769 (0.751 - 0.787)
Llama3-OpenBioLLM-8B	10	0.872 (0.856 - 0.886)	0.965 (0.956 - 0.973)	0.822 (0.779 - 0.873)	0.898 (0.863 - 0.934)	0.687 (0.659 - 0.723)	0.954 (0.921 - 0.986)	0.884 (0.871 - 0.897)	0.836 (0.827 - 0.843)	0.791 (0.772 - 0.812)
Llama-3.3-70B-Instruct	10	0.808 (0.772 - 0.836)	0.974 (0.954 - 0.993)	**1.000 (1.000 - 1.000)**	0.953 (0.912 - 0.988)	0.607 (0.569 - 0.647)	0.955 (0.875 - 1.000)	0.891 (0.863 - 0.919)	0.965 (0.954 - 0.975)	0.793 (0.758 - 0.819)
DeepSeek-R1-Distill-Llama-70B	0	0.844 (0.832 - 0.858)	0.992 (0.988 - 0.997)	0.947 (0.907 - 0.983)	0.929 (0.906 - 0.950)	0.660 (0.635 - 0.685)	0.987 (0.973 - 0.998)	0.965 (0.958 - 0.972)	0.964 (0.960 - 0.969)	0.831 (0.814 - 0.847)
gpt-4o-2024-08-06	0	0.952 (0.942 - 0.961)	0.993 (0.988 - 0.997)	0.945 (0.904 - 0.980)	0.955 (0.939 - 0.970)	0.630 (0.609 - 0.649)	0.969 (0.941 - 0.989)	0.983 (0.978 - 0.988)	0.967 (0.963 - 0.971)	0.857 (0.837 - 0.875)
o3-mini-2025-01-31	5	0.982 (0.975 - 0.988)	0.996 (0.992 - 0.998)	0.972 (0.946 - 0.992)	**0.969 (0.950 - 0.985)**	0.669 (0.643 - 0.694)	0.984 (0.962 - 0.998)	**0.992 (0.989 - 0.996)**	0.952 (0.947 - 0.958)	0.893 (0.881 - 0.902)
Llama-DrugDetector-70B	0	0.913 (0.902 - 0.924)	**0.996 (0.992 - 0.999)**	0.983 (0.961 - 0.999)	0.962 (0.944 - 0.979)	0.744 (0.713 - 0.781)	**0.989 (0.978 - 1.000)**	0.988 (0.983 - 0.993)	**0.978 (0.974 - 0.982)**	0.894 (0.882 - 0.907)
Llama-DrugDetector-8B	0	**0.988 (0.983 - 0.992)**	0.994 (0.990 - 0.998)	0.950 (0.919 - 0.982)	0.886 (0.862 - 0.909)	**0.939 (0.881 - 0.982)**	0.977 (0.957 - 0.995)	0.988 (0.985 - 0.993)	0.975 (0.971 - 0.979)	**0.929 (0.908 - 0.946)**

Table 10: Mean Positive Predictive Value (precision) on the held-out test set (n=6443) with bootstrapped lower and upper bounds for each detector and drug class. Abbreviations: Methamphetamine (Meth.), Benzodiazepine (Benzo.), Prescription Opioid Misuse (Rx. Opioids), Injection Drug Use (IDU).

Detector	Shots	Heroin	Cocaine	Meth.	Benzos.	Rx. Opioids	Cannabis	IDU	Any	Overall
Llama3-OpenBioLLM-8B	0	0.977 (0.973 - 0.981)	0.990 (0.987 - 0.993)	0.998 (0.997 - 0.999)	0.980 (0.977 - 0.984)	0.989 (0.986 - 0.991)	0.994 (0.993 - 0.996)	0.950 (0.945 - 0.955)	0.695 (0.681 - 0.710)	0.947 (0.944 - 0.949)
Bio_ClinicalBERT	0	0.960 (0.955 - 0.964)	0.918 (0.912 - 0.926)	0.989 (0.986 - 0.991)	0.964 (0.960 - 0.969)	0.981 (0.978 - 0.984)	0.981 (0.977 - 0.984)	0.960 (0.955 - 0.965)	0.973 (0.967 - 0.978)	0.966 (0.964 - 0.967)
bert-base-uncased	0	0.957 (0.953 - 0.963)	0.917 (0.909 - 0.924)	0.989 (0.986 - 0.991)	0.964 (0.959 - 0.968)	0.982 (0.977 - 0.985)	0.981 (0.978 - 0.985)	0.984 (0.981 - 0.988)	0.974 (0.968 - 0.980)	0.969 (0.967 - 0.971)
biobert-v1.1	0	0.959 (0.954 - 0.963)	0.918 (0.912 - 0.924)	0.989 (0.987 - 0.991)	0.964 (0.960 - 0.968)	0.981 (0.978 - 0.984)	0.981 (0.978 - 0.984)	0.987 (0.983 - 0.990)	0.977 (0.973 - 0.982)	0.969 (0.968 - 0.971)
ClinicalBERT	0	0.986 (0.983 - 0.989)	0.917 (0.911 - 0.924)	0.989 (0.986 - 0.991)	0.964 (0.959 - 0.968)	0.981 (0.977 - 0.983)	0.983 (0.980 - 0.986)	0.978 (0.974 - 0.983)	0.968 (0.963 - 0.975)	0.971 (0.969 - 0.972)
DeepSeek-R1-Distill-Llama-8B	10	0.996 (0.994 - 0.997)	0.995 (0.993 - 0.997)	0.999 (0.998 - 1.000)	0.990 (0.987 - 0.992)	0.992 (0.989 - 0.994)	0.997 (0.995 - 0.998)	0.969 (0.965 - 0.973)	0.845 (0.834 - 0.859)	0.973 (0.971 - 0.975)
o3-mini-2025-01-31	10	0.997 (0.995 - 0.998)	0.998 (0.997 - 0.999)	1.000 (0.999 - 1.000)	0.988 (0.986 - 0.990)	0.994 (0.992 - 0.996)	**1.000 (0.999 - 1.000)**	0.997 (0.995 - 0.998)	0.923 (0.914 - 0.931)	0.987 (0.986 - 0.988)
Llama3-Med42-8B	10	0.997 (0.996 - 0.999)	0.999 (0.998 - 1.000)	**1.000 (1.000 - 1.000)**	0.998 (0.997 - 0.999)	0.999 (0.998 - 1.000)	0.999 (0.998 - 1.000)	0.996 (0.994 - 0.997)	0.914 (0.905 - 0.924)	0.988 (0.987 - 0.989)
gpt-3.5-turbo-0125	10	0.994 (0.992 - 0.996)	0.998 (0.997 - 0.999)	1.000 (0.999 - 1.000)	0.998 (0.997 - 0.999)	0.996 (0.995 - 0.997)	0.999 (0.998 - 1.000)	0.991 (0.988 - 0.993)	0.938 (0.930 - 0.947)	0.989 (0.988 - 0.990)
Llama-3.1-8B-Instruct	10	0.995 (0.994 - 0.997)	0.997 (0.996 - 0.998)	0.998 (0.997 - 0.999)	0.995 (0.994 - 0.997)	**0.999 (0.999 - 1.000)**	0.999 (0.999 - 1.000)	0.996 (0.994 - 0.997)	0.934 (0.927 - 0.942)	0.989 (0.988 - 0.991)
DeepSeek-R1-Distill-Llama-70B	0	0.998 (0.997 - 0.999)	0.999 (0.998 - 1.000)	0.999 (0.998 - 0.999)	0.999 (0.997 - 0.999)	0.999 (0.998 - 1.000)	**1.000 (0.999 - 1.000)**	0.998 (0.996 - 0.999)	0.934 (0.925 - 0.942)	0.991 (0.990 - 0.992)
gpt-4o-2024-08-06	3	0.995 (0.993 - 0.997)	0.998 (0.997 - 0.999)	**1.000 (1.000 - 1.000)**	0.998 (0.997 - 0.999)	0.999 (0.998 - 1.000)	0.999 (0.999 - 1.000)	0.997 (0.995 - 0.998)	0.952 (0.944 - 0.959)	0.992 (0.991 - 0.993)
Llama-DrugDetector-70B	0	0.998 (0.997 - 0.999)	0.999 (0.998 - 0.999)	0.999 (0.998 - 0.999)	0.998 (0.997 - 0.999)	0.999 (0.997 - 0.999)	**1.000 (0.999 - 1.000)**	0.998 (0.997 - 0.999)	0.959 (0.953 - 0.966)	0.994 (0.993 - 0.995)
Llama-DrugDetector-8B	0	0.998 (0.998 - 0.999)	0.999 (0.998 - 1.000)	0.998 (0.997 - 0.999)	0.999 (0.998 - 1.000)	0.986 (0.983 - 0.989)	0.999 (0.999 - 1.000)	0.995 (0.993 - 0.997)	0.982 (0.977 - 0.987)	0.995 (0.994 - 0.995)
MedLlama3-8B	10	0.999 (0.997 - 1.000)	0.999 (0.998 - 1.000)	0.999 (0.999 - 1.000)	0.997 (0.996 - 0.998)	0.996 (0.994 - 0.998)	0.999 (0.998 - 0.999)	0.996 (0.993 - 0.999)	0.980 (0.961 - 0.991)	0.996 (0.993 - 0.997)
Llama-3.1-70B-Instruct	0	**0.999 (0.998 - 1.000)**	0.999 (0.998 - 1.000)	0.999 (0.998 - 1.000)	1.000 (0.999 - 1.000)	**0.999 (0.999 - 1.000)**	**1.000 (0.999 - 1.000)**	0.999 (0.998 - 1.000)	0.985 (0.980 - 0.988)	0.997 (0.997 - 0.998)
Llama-3.3-70B-Instruct	0	**0.999 (0.998 - 1.000)**	1.000 (0.999 - 1.000)	1.000 (0.999 - 1.000)	**1.000 (1.000 - 1.000)**	**0.999 (0.999 - 1.000)**	**1.000 (0.999 - 1.000)**	**0.999 (0.999 - 1.000)**	0.982 (0.978 - 0.987)	0.997 (0.997 - 0.998)
Llama3-Med42-70B	10	0.998 (0.997 - 0.999)	0.999 (0.999 - 1.000)	**1.000 (1.000 - 1.000)**	1.000 (0.999 - 1.000)	**0.999 (0.999 - 1.000)**	**1.000 (0.999 - 1.000)**	**1.000 (0.999 - 1.000)**	0.990 (0.986 - 0.993)	0.998 (0.998 - 0.999)
Llama3-OpenBioLLM-70B	5	0.999 (0.998 - 0.999)	**1.000 (1.000 - 1.000)**	**1.000 (1.000 - 1.000)**	0.999 (0.999 - 1.000)	**0.999 (0.999 - 1.000)**	**1.000 (0.999 - 1.000)**	**1.000 (0.999 - 1.000)**	**0.994 (0.991 - 0.996)**	**0.999 (0.998 - 0.999)**

Table 11: Mean Negative Predictive Value on the held-out test set (n=6443) with bootstrapped lower and upper bounds for each detector and drug class. Abbreviations: Methamphetamine (Meth.), Benzodiazepine (Benzo.), Prescription Opioid Misuse (Rx. Opioids), Injection Drug Use (IDU).

Detector	Shots	Heroin	Cocaine	Meth.	Benzos.	Rx. Opioids	Cannabis	IDU	Any	Overall
MedLlama3-8B	5	0.257 (0.246 - 0.266)	0.841 (0.832 - 0.850)	0.935 (0.929 - 0.943)	0.935 (0.930 - 0.941)	0.834 (0.825 - 0.843)	0.876 (0.867 - 0.886)	0.348 (0.334 - 0.360)	0.195 (0.182 - 0.206)	0.653 (0.647 - 0.659)
Llama3-Med42-70B	0	0.842 (0.831 - 0.851)	0.946 (0.942 - 0.951)	0.975 (0.972 - 0.979)	0.966 (0.961 - 0.969)	0.905 (0.897 - 0.911)	0.958 (0.953 - 0.962)	0.785 (0.775 - 0.797)	0.886 (0.877 - 0.896)	0.908 (0.904 - 0.912)
Llama3-Med42-8B	10	0.807 (0.797 - 0.820)	0.952 (0.947 - 0.958)	0.969 (0.965 - 0.974)	0.959 (0.954 - 0.963)	0.910 (0.904 - 0.917)	0.970 (0.967 - 0.975)	0.926 (0.918 - 0.933)	0.969 (0.964 - 0.976)	0.933 (0.929 - 0.938)
Llama3-OpenBioLLM-70B	10	0.871 (0.861 - 0.881)	0.979 (0.974 - 0.982)	0.983 (0.980 - 0.986)	0.971 (0.966 - 0.975)	0.909 (0.903 - 0.917)	0.985 (0.982 - 0.988)	0.956 (0.951 - 0.962)	0.944 (0.938 - 0.952)	0.950 (0.947 - 0.953)
biobert-v1.1	0	0.919 (0.913 - 0.926)	0.997 (0.996 - 0.998)	0.999 (0.999 - 1.000)	0.998 (0.996 - 0.999)	**0.999 (0.998 - 1.000)**	0.999 (0.998 - 1.000)	0.805 (0.795 - 0.814)	0.915 (0.908 - 0.923)	0.954 (0.952 - 0.956)
ClinicalBERT	0	0.928 (0.922 - 0.934)	0.994 (0.992 - 0.995)	0.996 (0.995 - 0.998)	0.998 (0.997 - 0.999)	0.998 (0.997 - 0.999)	0.991 (0.989 - 0.993)	0.810 (0.800 - 0.818)	0.920 (0.911 - 0.929)	0.954 (0.952 - 0.957)
Llama-3.1-8B-Instruct	0	0.930 (0.923 - 0.937)	0.986 (0.984 - 0.988)	0.977 (0.974 - 0.981)	0.975 (0.971 - 0.979)	0.873 (0.865 - 0.881)	0.992 (0.990 - 0.994)	0.929 (0.923 - 0.936)	0.993 (0.990 - 0.995)	0.957 (0.955 - 0.959)
bert-base-uncased	0	0.962 (0.957 - 0.966)	0.998 (0.997 - 0.999)	**1.000 (0.999 - 1.000)**	0.997 (0.996 - 0.999)	0.998 (0.997 - 0.999)	0.999 (0.998 - 1.000)	0.810 (0.800 - 0.820)	0.930 (0.921 - 0.939)	0.962 (0.959 - 0.964)
Bio_ClinicalBERT	0	0.942 (0.936 - 0.948)	0.997 (0.995 - 0.998)	0.999 (0.999 - 1.000)	0.995 (0.993 - 0.996)	0.997 (0.996 - 0.998)	0.997 (0.996 - 0.998)	0.861 (0.851 - 0.870)	0.905 (0.896 - 0.914)	0.962 (0.959 - 0.964)
Llama-3.3-70B-Instruct	0	0.910 (0.902 - 0.916)	0.997 (0.996 - 0.998)	0.997 (0.996 - 0.998)	0.990 (0.988 - 0.993)	0.926 (0.920 - 0.932)	0.997 (0.996 - 0.998)	0.944 (0.938 - 0.951)	0.977 (0.973 - 0.983)	0.967 (0.966 - 0.969)
Llama-3.1-70B-Instruct	0	0.943 (0.937 - 0.950)	0.998 (0.997 - 0.999)	0.998 (0.997 - 0.999)	0.989 (0.986 - 0.991)	0.929 (0.924 - 0.935)	0.998 (0.997 - 0.999)	0.949 (0.944 - 0.955)	0.969 (0.963 - 0.975)	0.972 (0.970 - 0.974)
gpt-3.5-turbo-0125	10	0.940 (0.934 - 0.947)	0.992 (0.990 - 0.994)	0.987 (0.984 - 0.990)	0.979 (0.976 - 0.983)	0.965 (0.960 - 0.969)	0.997 (0.995 - 0.998)	0.958 (0.952 - 0.964)	0.964 (0.959 - 0.970)	0.973 (0.971 - 0.975)
DeepSeek-R1-Distill-Llama-8B	5	0.940 (0.934 - 0.945)	0.982 (0.978 - 0.985)	0.988 (0.986 - 0.991)	0.987 (0.984 - 0.990)	0.978 (0.975 - 0.982)	0.991 (0.988 - 0.993)	0.959 (0.953 - 0.963)	0.962 (0.957 - 0.968)	0.974 (0.971 - 0.976)
DeepSeek-R1-Distill-Llama-70B	5	0.935 (0.929 - 0.942)	0.995 (0.993 - 0.996)	0.996 (0.994 - 0.997)	0.998 (0.997 - 0.999)	0.972 (0.968 - 0.976)	0.999 (0.998 - 0.999)	0.993 (0.991 - 0.995)	0.995 (0.993 - 0.997)	0.985 (0.984 - 0.987)
gpt-4o-2024-08-06	0	0.987 (0.984 - 0.990)	0.999 (0.998 - 1.000)	0.999 (0.998 - 1.000)	0.996 (0.995 - 0.998)	0.950 (0.945 - 0.954)	0.999 (0.998 - 1.000)	0.994 (0.992 - 0.996)	0.991 (0.987 - 0.993)	0.989 (0.988 - 0.990)
Llama3-OpenBioLLM-8B	10	0.978 (0.974 - 0.981)	0.997 (0.995 - 0.998)	0.996 (0.994 - 0.997)	0.995 (0.994 - 0.997)	0.985 (0.982 - 0.987)	0.999 (0.999 - 1.000)	0.982 (0.978 - 0.985)	0.987 (0.982 - 0.990)	0.990 (0.989 - 0.991)
Llama-DrugDetector-70B	0	0.973 (0.969 - 0.977)	0.999 (0.999 - 1.000)	**1.000 (0.999 - 1.000)**	0.997 (0.996 - 0.999)	0.981 (0.978 - 0.985)	**1.000 (0.999 - 1.000)**	0.996 (0.994 - 0.997)	**0.997 (0.995 - 0.999)**	0.993 (0.992 - 0.994)
Llama-DrugDetector-8B	3	0.993 (0.991 - 0.995)	0.999 (0.998 - 0.999)	0.999 (0.998 - 0.999)	0.992 (0.990 - 0.994)	0.996 (0.995 - 0.998)	0.999 (0.998 - 1.000)	0.997 (0.996 - 0.999)	0.978 (0.973 - 0.982)	0.994 (0.993 - 0.995)
o3-mini-2025-01-31	10	**0.996 (0.994 - 0.997)**	**1.000 (0.999 - 1.000)**	0.999 (0.998 - 1.000)	**0.999 (0.998 - 1.000)**	0.973 (0.969 - 0.976)	0.999 (0.999 - 1.000)	**0.998 (0.997 - 0.999)**	0.992 (0.988 - 0.995)	**0.994 (0.994 - 0.995)**

Table 12: Mean Specificity on the held-out test set (n=6443) with bootstrapped lower and upper bounds for each detector and drug class. Abbreviations: Methamphetamine (Meth.), Benzodiazepine (Benzo.), Prescription Opioid Misuse (Rx. Opioids), Injection Drug Use (IDU).

Appendix F. Drug Co-occurences

Drugs	Heroin	Cocaine	Meth.	Benzo.	Rx. Opioids	Cannabis	IDU
**Heroin**	749	196	18	43	26	32	254
**Cocaine**	196	528	20	56	11	44	120
**Meth.**	18	20	72	11	1	3	21
**Benzo.**	43	56	11	232	28	13	21
**Rx. Opioids**	26	11	1	28	122	5	22
**Cannabis**	32	44	3	13	5	121	14
**IDU**	254	120	21	21	22	14	1041

Table 13: Co-occurence of substances in the test-split of the DrugDetection dataset. Abbreviations: Methamphetamine (Meth.), Benzodiazepine (Benzo.), Prescription Opioid Misuse (Rx. Opioids), Injection Drug Use (IDU).

Table 13 presents the co-occurrence matrix of various substances within the test split of the DrugDetection dataset, shedding light on the relationships between different drugs. The diagonal entries indicate the frequency of individuals using each specific substance, with injecting drug use (IDU) being the most common at 1041 occurrences. Significant co-occurrences are observed between heroin and cocaine (196 cases) and between heroin and IDU (254 cases), reflecting a notable overlap among these substances. This table highlights the prevalence of polysubstance use within the dataset, particularly with regard to IDU, heroin, and cocaine.

Appendix G. ICD Code Analysis Tables

Category	ICD-9 Codes	ICD-10 Codes
Heroin	96501, 30400, 30401, 30402, 30550, 30551, 30552, 96500, 96502, 96509	F11
Cocaine	30420, 30421, 30422, 30560, 30561, 30562, 97081	F14
Methamphetamine	30440, 30441, 30442, 30443, 30570, 30571, 30572, 96972	F15
Benzodiazepine	30410, 30411, 30412, 30540, 30541, 30542, 9694	F13
Rx Opioid Misuse	30400, 30401, 30402, 30550, 30551, 30552, 96500, 96502, 96509	F11
Cannabis	30430, 30431, 30432, 30520, 30521, 30522	F12
Injection Drug Use	–	–
General Drug Use	30490, 30491, 30492, 30493, 30590, 30591, 30592, 30593	F19, Z72, Z86

Table 14: Mapping between substance categories and the ICD-9/ICD-10 codes used for labeling. ICD-9 lists enumerate specific codes; ICD-10 entries denote code *prefixes* (e.g., `F11` indicates the `F11.*` block), not exhaustive subcode lists. Heroin shares the Rx Opioid Misuse code set. "Injection drug use" is intentionally left unspecified because no dedicated codes are included in this mapping. "General drug use" captures non–substance-specific diagnoses (ICD-10 prefixes `F19`, `Z72`, `Z86`) as well as all other codes in the table.

Metric	Heroin	Cocaine	Meth.	Benzo.	Rx. Opioids	Cannabis	Any
# Known Use	382	237	44	136	98	83	690
# ICD Codes	209	102	14	26	55	20	406
Sensitivity	54.70%	43.00%	31.80%	19.10%	56.10%	24.10%	58.80%
Specificity	87.80%	96.60%	99.50%	98.80%	77.40%	98.30%	91.40%
F1 Score	60.80%	55.00%	43.80%	29.70%	27.70%	32.80%	71.40%

Table 15: Patient-level performance of ICD-based identification of substance use by category. "# Known Use" counts patients documented as using each substance via human annotation; "# ICD Codes" counts patients with *any* corresponding ICD-9/ICD-10 code. Sensitivity, specificity, and F1 are computed using Known Use as the reference and the presence of any related code as positive. "Injection Drug Use" is excluded due to a lack of corresponding ICD-9/10 codes. Abbreviations: Methamphetamine (Meth.), Benzodiazepine (Benzo.), Prescription Opioid Misuse (Rx. Opioids)

Appendix H. Racial Fairness Analysis Figures

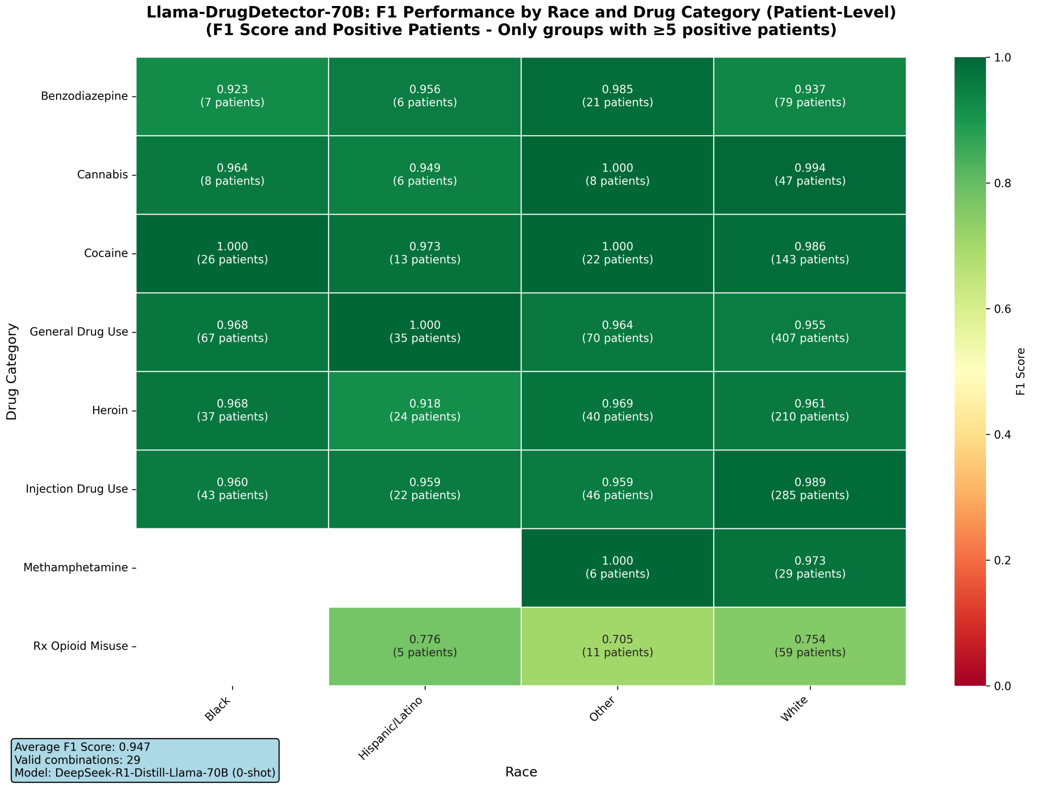

Figure 3: Per-race F1 performance for the `Llama-DrugDetector-70B` model. Cell labels show the F1-score and the number of positive patients in each subgroup.

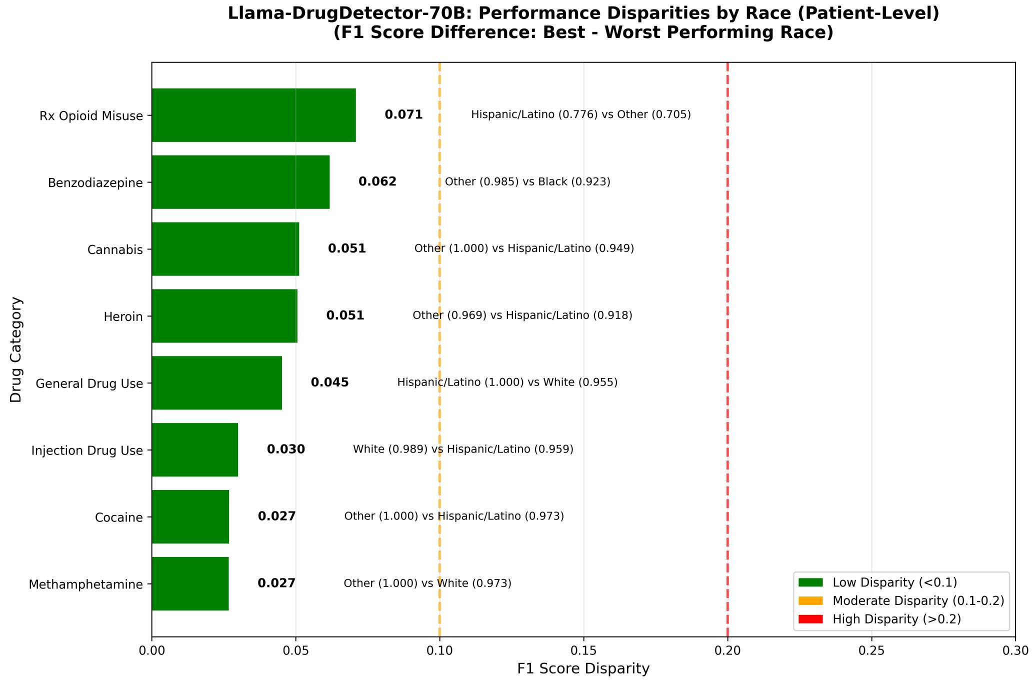

Figure 4: F1 disparity by substance, showing the absolute difference between the best- and worst-performing racial groups. All disparities fall below the 0.1 threshold, indicating low disparity.
